# Development of Fluorine‐Free Tantalum Carbide MXene Hybrid Structure as a Biocompatible Material for Supercapacitor Electrodes

**DOI:** 10.1002/adfm.202100015

**Published:** 2021-05-24

**Authors:** Alireza Rafieerad, Ahmad Amiri, Glen Lester Sequiera, Weiang Yan, Yijun Chen, Andreas A. Polycarpou, Sanjiv Dhingra

**Affiliations:** ^1^ Regenerative Medicine Program Institute of Cardiovascular Sciences St. Boniface Hospital Research Centre Department of Physiology and Pathophysiology Rady Faculty of Health Sciences University of Manitoba Winnipeg MB R2H 0G1 Canada; ^2^ J. Mike Walker '66 Mechanical Engineering Department Texas A&M University College Station TX 77843 USA; ^3^ Department of Aerospace Engineering Texas A&M University College Station TX 77843 USA

**Keywords:** biocompatible electrode, fluorine‐free Ta _4_C _3_T*_x_* MXene, human stem cells, hybrid structures, supercapacitors, Ta _4_C _3_T*_x_* MXene‐tantalum oxide

## Abstract

The application of nontoxic 2D transition‐metal carbides (MXenes) has recently gained ground in bioelectronics. In group‐4 transition metals, tantalum possesses enhanced biological and physical properties compared to other MXene counterparts. However, the application of tantalum carbide for bioelectrodes has not yet been explored. Here, fluorine‐free exfoliation and functionalization of tantalum carbide MAX‐phase to synthesize a novel Ta_4_C_3_T_x_ MXene‐tantalum oxide (TTO) hybrid structure through an innovative, facile, and inexpensive protocol is demonstrated. Additionally, the application of TTO composite as an efficient biocompatible material for supercapacitor electrodes is reported. The TTO electrode displays long‐term stability over 10 000 cycles with capacitance retention of over 90% and volumetric capacitance of 447 F cm^−3^ (194 F g^−1^) at 1 mV s^−1^. Furthermore, TTO shows excellent biocompatibility with human‐induced pluripotent stem cells‐derived cardiomyocytes, neural progenitor cells, fibroblasts, and mesenchymal stem cells. More importantly, the electrochemical data show that TTO outperforms most of the previously reported biomaterials‐based supercapacitors in terms of gravimetric/volumetric energy and power densities. Therefore, TTO hybrid structure may open a gateway as a bioelectrode material with high energy‐storage performance for size‐sensitive applications.

## Graphical abstract

A new fluorine‐free tantalum carbide MXene‐tantalum oxides (TTO) nanostructure is developed as a biocompatible electrode material for size‐sensitive applications. The TTO hybrid structure is biocompatible with different types of human cells, and offers excellent volumetric capacitance, energy density, power density, and cyclability when assembled into a symmetric supercapacitor. The TTO offers high promise for future biomedical energy storage devices.

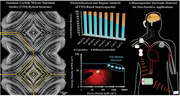

## 1 Introduction

With ongoing rapid development in the area of bioelectronics, small and light weight biocompatible electrodes are in high demand for biomedical implantable devices such as, cardiac pacemakers, neurostimulators, and cochlear implants.^[^ ^1^, ^2^, ^3^, ^4^, ^5^ ^]^ The supercapacitors (SCs) with promising properties, such as, high power density, unlimited cycle life, eco‐friendliness, and low‐temperature charging ability, can become the ideal energy storage devices for medical electronics. Furthermore, implantable SCs can also be used for monitoring biological and electrophysiological information inside the human and mammalian body. Therefore, an ideal SC should be biocompatible, miniature in size, and it should possess high energy and power densities. Additionally, the electrochemical stability of biocompatible SC‐based systems under physiological conditions is another advantage. However, current SCs consisting of biocompatible electrode materials are not able to meet all the aforementioned requirements in a single package. To date, several studies have reported the fabrication of biocompatible energy storage devices using advanced carbon‐based materials such as, graphene nanosheets, carbon nanotubes, and fibers with a focus on maximizing the electric double layer (EDL) capacitive properties.^[^ ^6^, ^7^, ^8^ ^]^ However, bioelectrical applications of these materials are limited in terms of the energy surface support including low energy density per unit volume or mass.^[^ ^9^ ^]^ It is important to further note that these anisotropic carbon structures implicate decreased biocompatibility in terms of cellular growth, proliferation, and differentiation.^[^ ^10^ ^]^

In this regard, recently reported 2D transition metal carbides and nitrides (MXenes) are considered among booming materials because of their application in multiple fields.^[^ ^11^, ^12^, ^13^, ^14^ ^]^ The main energy‐storage characteristics of the MXene nanosheets are excellent volumetric capacitance and energy density, which are vital for size‐sensitive applications. MXene materials possess hydrophilic surfaces and are selectively etched from their MAX phase structures, where “M” represents one of the early transition metals (e.g., titanium, tantalum, niobium, or zirconium), “A” denotes one of the A‐group elements (e.g., aluminum, silicon, or phosphorus) and “X” denotes either carbon or nitrogen. MXene structures possess tremendous physicochemical, electrical, optical and biological properties which have enabled them to be exquisitely used in various electrical and biological applications.^[^ ^15^, ^16^, ^17^ ^]^ Given this, MXene is a suitable class of material for preparing electrodes for energy storage due to its superior metallic conductivity, electrochemical stability and higher volumetric capacitance compared to the conventional carbon‐based electrode materials.^[^ ^13^, ^18^ ^]^

In previous studies, the exfoliation of different forms of MXene such as titanium carbide (Ti_3_C_2_T*_x_*) and niobium carbide (Nb_2_CT*_x_*) has been reported for application in lithium‐ion (Li‐ion) batteries, capacitors and regenerative medicine.^[^ ^19^, ^20^, ^21^ ^]^ Recently, a new composition of MXene nanosheets, tantalum carbide (Ta_4_C_3_T*_x_*) has been reported.^[^ ^22^, ^23^ ^]^ Tantalum (Ta)‐based materials are well‐known due to their excellent bio‐functionality when compared to other MXene counterparts such as titanium (Ti)‐ and niobium (Nb)‐based composites.^[^ ^24^, ^25^, ^26^, ^27^ ^]^ Moreover, the physical properties of Ta, including density, electrical conductivity and mechanical Young's modulus are higher in comparison to Ti and Nb. In fact, the exfoliated tantalum carbide MXene nanosheets have been recently reported as efficient nanoplatforms for cancer therapy.^[^ ^28^ ^]^ However, in most of the MXene based previous reports, hydrofluoric acid (HF) was used as an etchant to remove aluminum (Al) from the MAX phase. Unfortunately, HF is highly corrosive and causes significant burns and toxicity upon contact, ingestion, or inhalation. More importantly, it leads to the formation of fluorine bonds in the end product that substantially decreases the electrochemical activity of MXene‐based electrodes. Additionally, the application of fluorine‐containing etchants leads to interaction between the remaining Al from the MAX phase structure and inert fluorine terminals, which is environmentally harmful during application of MXene‐based materials. Therefore, fluorine containing etchants could potentially decrease the biocompatibility and volumetric capacitance properties of MXene‐based energy storage electrodes.

Furthermore, functionalization of oxygen groups in the structure of 2D MXene nanosheets is reported to enhance the electrochemical performance of the composite.^[^ ^18^ ^]^ The available literature on oxidized Ti_3_C_2_T*_x_* and Nb_2_CT*_x_* MXene composites revealed that the formation of crystalline transition metal oxide particles up to a few hundred nanometers in size enhanced the surface activity of nanosheets.^[^ ^13^, ^29^, ^30^ ^]^ Therefore, it is conceptualized that the formation of oxygen‐containing functional groups on the surface of Ta_4_C_3_T*_x_* will promote its electrochemical properties when used as supercapacitor electrode. Furthermore, growth of metal oxide crystals on the surface of MXene prevents restacking of exfoliated MXene due to van der Waals interaction; therefore, functionalization of oxygen‐containing groups preserves the electrochemical performance of delaminated MXene.

In the current study, we present, for the first time, oxidized fluorine‐free exfoliation of tantalum carbide MAX phase to synthesize a new Ta_4_C_3_T*_x_* MXene‐tantalum oxide (TTO) hybrid structure. Our study demonstrates that TTO has excellent potential to be used as a bioelectrode material for long‐term supercapacitor applications. This new electrode has long‐term electrochemical stability, excellent volumetric capacitance, and high energy/power densities and charging rate. Our findings confirm that the TTO hybrid structure is highly biocompatible with different human cell types. The energy density of the TTO electrode outperforms almost all the existing biomaterial‐based electrodes. In addition, volumetric capacitance of the TTO is significantly higher than the majority of previously reported organic/inorganic biocompatible electrodes. This novel TTO nanostructure may act as a favorite electrode material for future applications in size‐sensitive biomedical energy storage devices.

## 2 Results and Discussion

### 2.1 Fabrication of Ta_4_C_3_T*_x_* MXene‐Tantalum Oxide Hybrid Structure

**Figure 1:**
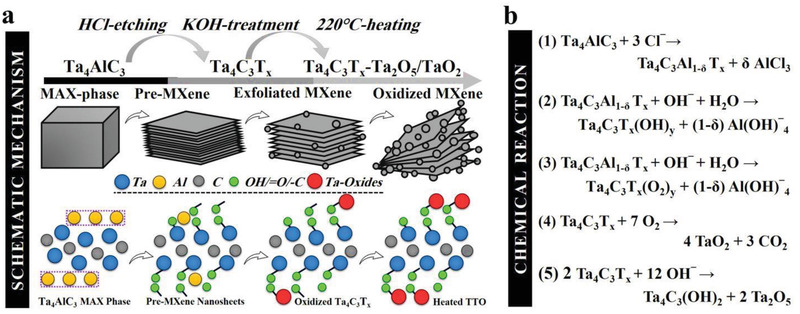
Schematic model and stoichiometry of TTO hybrid structure. Illustration of a) step‐by‐step schematic and b) mechanism of reaction for the fluorine‐free conversion of the Ta_4_AlC_3_ MAX phase to surface‐modified Ta_4_C_3_T*_x_* MXene nanosheets decorated with tantalum oxide nanoparticles.

We employed an innovative fluorine‐free etching method to prepare TTO hybrid nanostructure from raw and bulk material. Recently, application of an alkaline‐induced method for removal of Al from the Ti_3_AlC_2_ MAX phase to synthesize Ti_3_C_2_T*_x_* MXene was reported.^[^ ^31^ ^]^ However, the main challenge in etching the Al layer from MAX phase in alkaline media is the blocked/slow kinetic reactions due to the formation of unwanted oxide/hydroxide layers on the MXene surface.^[^ ^32^ ^]^ To address this, we utilized a modified alkaline‐based etching method to prepare the TTO via a two‐step acidic/alkaline (HCl/KOH) treatment. Briefly, Ta_4_AlC_3_ MAX phase powder was treated sequentially with 6 m hydrochloric acid (HCl) solution and 6 m potassium hydroxide (KOH) solution to synthesize an exfoliated nanocomposite. This led to formation of multilayered oxidized Ta_4_C_3_T*_x_* nanosheets anchored with Ta‐oxide particles. These were subsequently subjected to thermal treatment at 220 °C under moderate air heating for further functionalization and oxidation, resulting in the formation of final TTO hybrid structure. The step‐by‐step schematic model for the synthesis and functionalization of the mixed‐dimensional TTO nanocomposite is shown in **Figure** **1**a.

In our proposed mechanism of reaction (Figure 1b), the Al‐atoms on the edge and outer surface of Ta_4_AlC_3_ MAX phase were rapidly chlorinated using HCl through the production of soluble aluminum chloride (AlCl_3_).^[^ ^31^ ^]^ Therefore, a considerable amount of surface Al atoms are thought to be removed during this step. Subsequent etching treatment in KOH solution further led to lower levels of insoluble aluminum hydroxide [Al(OH)_3_] and aluminum oxide hydroxide [AlO(OH)] on the surface of the material when compared to the classic alkaline protocol. The lattice‐like features of the Ta‐layers limit the transformation of insoluble Al‐based compounds to soluble aluminate [Al(OH)_4_^−^].^[^ ^31^, ^32^ ^]^ However, the hybrid protocol employed in this study allows continued exfoliation and secondary crystal nucleation of the tantalum carbide material to give rise to oxidized Ta_4_C_3_T*_x_* MXene nanosheets. Furthermore, ongoing oxidation of exposed inner Al and Ta atoms by OH^−^ resulted in a higher degree of functionalization with —OH and = O groups.

### 2.2 Characterization of Ta_4_C_3_T*_x_* MXene‐Tantalum Oxide Hybrid Structure

**Figure 2:**
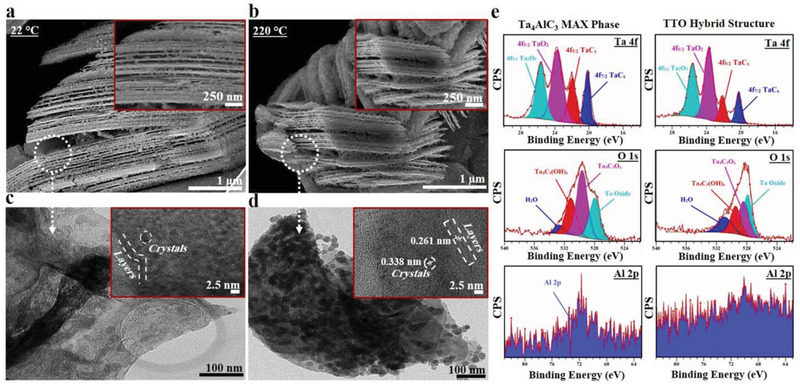
Morphology and microstructural characterization of the synthesized TTO hybrid structure. Scanning electron microscopic (SEM) images of the functionalized a) Ta_4_C_3_T*_x_* MXene and b) TTO nanostructure samples after heat treatment at 220 °C for 2 h. Field‐emission SEM observations of TTO hybrid structure reveal effective delamination of layers, anchored by a tantalum oxide particle array. The thermal treatment further improved the oxidization of Ta_4_C_3_T*_x_* MXene layers. TEM images of the oxidized c) Ta_4_C_3_T*_x_* MXene (inset) and d) TTO (inset) composites. The images show successful organization of TTO hybrid structures. The thermal treatment led to further improvement and well‐defined distribution of Ta‐oxide nanoparticles. High‐resolution TEM images confirmed the presence of two different lattices with d‐spacing of 0.261 and 0.338 nm, which is attributed to Ta_4_C_3_T*_x_* MXene layers and tantalum oxide composites. e) XPS narrow scan spectra of Ta 4f, O 1s, Al 2p corresponding to Ta_4_AlC_3_ MAX phase and oxidized TTO samples after thermal treatment at 220 °C for 2 h, confirming proper extraction of Al from the MAX phase structure with effective exfoliation of MXene nanosheets. The XPS fittings further demonstrate that exfoliated Ta_4_C_3_T*_x_* MXene sheets were successfully composited with Ta_2_O_5_‐TaO_2_ particles.

The scanning electron microscopic (SEM) images of the functionalized Ta_4_C_3_T*_x_* MXene nanosheets prior to thermal treatment are presented in **Figure** **2**a. The well‐exfoliated MXene nanosheets are strewn with a considerable number of tantalum oxide nanoparticle clusters. Subsequent thermal treatment at 220 °C for 2 h led to significantly enhanced exfoliation and functionalization of MXene nanosheets with Ta‐oxide nanoparticles, which is described here as a TTO hybrid structure (Figure 2b and Figure S1, Supporting Information). Furthermore, field‐emission SEM images of the oxidized Ta_4_C_3_T_x_ MXene and TTO hybrid structure revealed a slight decrease in the wall‐to‐wall interlayer space of MXene nanosheets after thermal treatment (Figure S2a,b, Supporting Information).

The high‐resolution transmission electron microscopy (TEM) of oxidized Ta_4_C_3_T*_x_* MXene and the TTO hybrid structure revealed that individual nanoparticles are ≈5 nm in diameter and cluster to form larger decorations seen in the SEM images (Figure 2c,d). The fast Fourier transform (FFT) analysis of TTO hybrid structure showed d‐spacing lattices of 0.261 and 0.368 nm, corresponding to oxidized Ta_4_C_3_T*_x_* MXene and tantalum oxide (Ta_2_O_5_) crystals respectively (Figure 2d and inset). These lattice parameters are in good agreement with previously reported literature on stable Ta_4_C_3_T*_x_* MXene and Ta_2_O_5_.^[^ ^28^, ^33^, ^34^, ^35^ ^]^ This FFT analysis is also congruent with published literature on the coexistence of bulky tantalum carbide (TaC) and Ta_2_O_5_, which reported lattice spacing of ≈0.26 and 0.38 nm, corresponding to the TaC (111) and Ta_2_O_5_ (001) planes, respectively.^[^ ^33^, ^34^, ^35^ ^]^

The selected area electron diffraction (SAED) patterns of TTO hybrid structure displayed a higher degree of crystalline structure with well‐defined hexagonal planes compared to oxidized Ta_4_AlC_3_ MXene samples (Figure S2c,d, Supporting Information). Together these observations provide robust evidence that the innovative fluorine‐free exfoliation and functionalization protocol employed in the current study has worked successfully to synthesize TTO nanostructure from the Ta_4_AlC_3_ MAX phase (Figure S3, Supporting Information).

The physicochemical properties of materials were further evaluated by X‐ray photoelectron spectroscopy (XPS), energy‐dispersive X‐ray spectroscopy (EDS), SAED, and X‐ray diffraction (XRD) analysis. XPS was used to characterize the structural transformation of Ta_4_AlC_3_ MAX phase to oxidized Ta_4_C_3_T*_x_* MXene and TTO hybrid structure. Comparison of survey spectra showed a significant change in the elemental composition of materials during the synthesis process (Figure S4a, Supporting Information). In particular, characteristic Al 2p peaks of Ta_4_AlC_3_ MAX phase were significantly decreased in the TTO hybrid structure. High resolution XPS spectra of TTO hybrid structure showed well‐defined characteristics of Ta_4_C_3_T*_x_* MXene. A comparison between Al 2p spectra of the Ta_4_AlC_3_ MAX phase and TTO confirmed effective elimination of Al layer, with more than 84% of elemental Al removed during the hybrid synthesis process (Figure 2e, Figure S5 and Table S1, Supporting Information). This degree of exfoliation is highly comparable with the widely‐used HF etching method for exfoliation.^[^ ^22^ ^]^ This is confirmed by the spectra of Ta 4f, which revealed two main peaks of TaC (4f 5/2 and 4f 7/2) at binding energies of 18 and 24 eV attributed to exfoliated Ta_4_C_3_T*_x_* MXene nanosheets.

The Ta 4f, O 1s, and C 1s spectra demonstrated a change in the degree of exfoliation and functionalization when converting Ta_4_AlC_3_ MAX phase to TTO hybrid structure (Figure 2e, Figure S4b–d and Table S1, Supporting Information). Ta_4_C_3_O*_x_* decreased from 41.7% to 27.6%, and Ta_4_C_3_(OH)*_x_* increased from 30.8% to 31.6%. Additionally, these spectra revealed an approximately 20% decrease in the surface atomic ratio of Ta‐C bonds and an increase of 10.43% in the surface atomic ratio of Ta‐O bonds in the TTO hybrid structure. The TTO hybrid structure contains two lateral species of Ta^4+^ and Ta^5+^ as the main tantalum oxide crystals of Ta_2_O_5_ and TaO_2_ at the binding energy of 22 to 27 eV in the Ta 4f spectrum. Taken together, these findings confirm the formation of tantalum oxide during the synthetic process.

Finally, the successful synthesis of TTO hybrid structure using the fluorine‐free process was further corroborated by EDS and XRD. The EDS elemental analysis demonstrated successful extraction of Al from the structure of Ta_4_AlC_3_ MAX phase with a decrease in the atomic percentage of Al from 20.57% to 11.31% (Figures S6 and S7, Supporting Information). The average weight percentage of Al similarly decreased from 12.64% to 4.43%. Concurrently, the atomic percentage of oxygen increased from 20.03% to 30.05%. The histogram also confirmed the absence of F, Cl, and K in the final composition of the TTO hybrid structure.

The XRD pattern of the Ta_4_AlC_3_ bulk material was typical with standard peaks at their expected 2θ values (Figure S8, Supporting Information).^[^ ^22^ ^]^ In agreement with the previous observations, peaks originating from the aluminum‐containing MAX phase were significantly decreased after fluorine‐free etching and exfoliation by the HCl/KOH process. One of the Ta_4_AlC_3_ peaks at around 16.5° 2θ was entirely removed in the TTO hybrid structure. Additionally a minor contamination peak ascribed to Ta_2_C with the reflection at 2θ ≈ 50° was absent in the XRD spectrum of TTO hybrid structure.^[^ ^36^ ^]^ Furthermore, a newly emerged (002) peak at around 7° 2θ corresponds to an aluminum‐etched tantalum carbide‐tantalum oxide material with the enlarged lattice parameters. Additional downshifts are also observed in the XRD spectra of the TTO hybrid structure due to increased carbon lattice spacing after the acid/alkaline treatment. Finally, characteristic small peaks corresponding to tantalum oxide particles anchored on the MXene surface were also observed in the structure of the TTO. Together these findings support the successful synthesis and functionalization of layered TTO hybrid structure using a fluorine‐free exfoliation and functionalization protocol.

### 2.3 Specific Surface Area of Ta_4_C_3_T*_x_* MXene‐Tantalum Oxide Hybrid Structure

**Figure 3:**
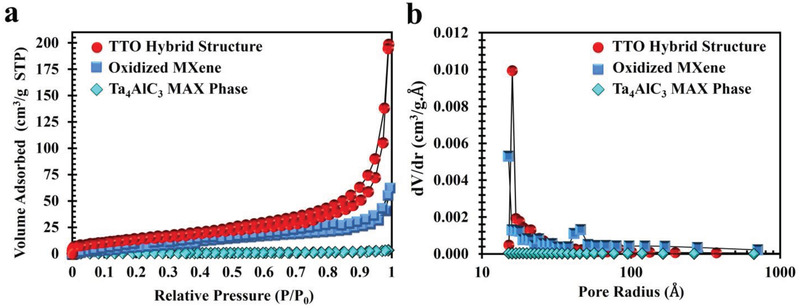
Specific surface area measurement using Brunauer–Emmett–Teller analysis. a) N_2_ adsorption‐desorption isotherm curves of the Ta_4_AlC_3_ MAX phase, oxidized Ta_4_C_3_T*_x_* MXene, and TTO hybrid structure. The BET data depicted that specific surface area of the materials was 1.29, 41.79, and 51.02 m^2^ g^−1^, respectively. b) Pore size distribution of the MAX phase, oxidized MXene, and TTO hybrid structure. As shown, the average pore diameter of the MAX phase was decreased about four‐fold in TTO nanostructure.

The surface area of carbon‐based nanomaterials is an important determinant of their electrochemical properties. The specific surface area of Ta_4_AlC_3_ MAX phase, oxidized Ta_4_C_3_T*_x_* MXene, and TTO hybrid structure was determined using Brunauer–Emmett–Teller (BET) nitrogen adsorption isotherms (**Figure** **3**a). The specific surface area of Ta_4_AlC_3_ MAX phase, oxidized Ta_4_C_3_T*_x_* MXene, and TTO hybrid structures were 1.29, 41.79, and 51.02 m^2^ g^−1^ respectively. There was a 40‐fold increase (approximately) in the surface area from Ta_4_AlC_3_ MAX phase to oxidized Ta_4_C_3_T*_x_* MXene, which is a result of Al etching and formation of a porous MXene structure.^[^ ^37^, ^38^, ^39^ ^]^ The specific surface area of the TTO hybrid structure is ≈20% higher than that of the oxidized Ta_4_C_3_T*_x_* MXene and can be attributed to higher levels of Ta‐oxide nanoparticles on the surface of the oxidized MXene material. This high surface area can be readily detected by assessing the optical properties of the TTO hybrid structure. The aqueous suspension of TTO hybrid structure (50 µg mL^−1^) exhibited high degrees of autoflorescence at several wavelengths across the visible spectrum (Figure S9, Supporting Information).

Consistent with Barrett‐Joyner‐Halenda theory, the total pore volume was increased by ≈14‐fold in the TTO hybrid structure when compared with Ta_4_AlC_3_ MAX phase. The average pore diameter, however, decreased from 81.15 nm in Ta_4_AlC_3_ MAX phase to 32.25 nm in oxidized Ta_4_C_3_T*_x_* MXene and 24.42 nm in TTO hybrid structure (Figure 3b). The dramatic increase in specific surface area during the synthesis process can thus be explained by a significant increase in the overall porosity of the TTO hybrid structure through formation of new micro‐ and mesopores during the hybrid acid/alkali and thermal treatments.

### 2.4 Electrochemical Properties of Ta_4_C_3_T*_x_* MXene‐Tantalum Oxide Hybrid Electrode

**Figure 4:**
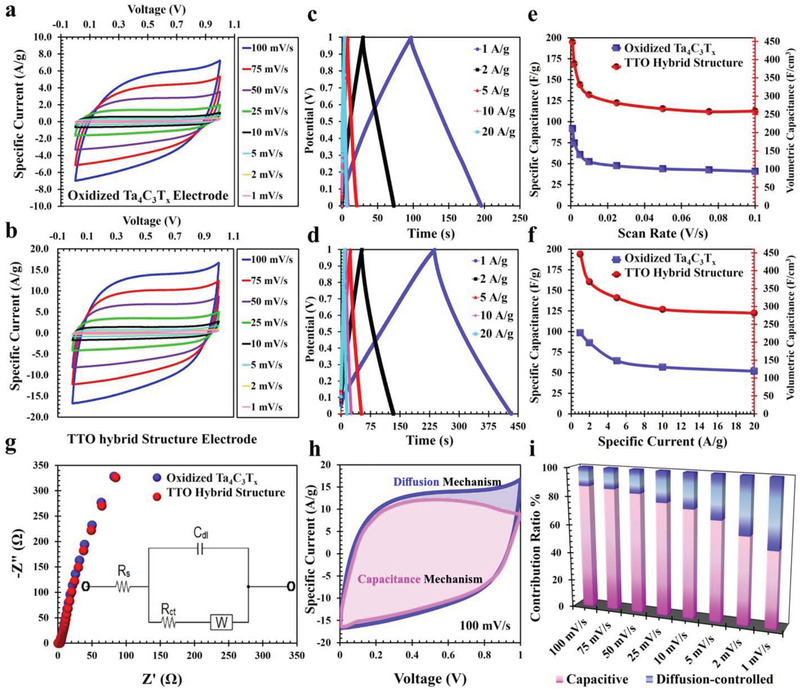
Electrical and electrochemical measurements of fabricated TTO hybrid structure electrode. a) Cyclic voltammetry curves of the oxidized Ta_4_C_3_T*_x_* MXene electrode and b) TTO hybrid structure electrode at different scan rates in PVA/H_3_PO_4_ solid electrolyte after 10 000 cycles of the two‐electrode experiment. c) The galvanostatic charge/discharge (GCD) curves of the oxidized Ta_4_C_3_T*_x_* MXene electrode and d) the TTO hybrid structure electrode. e) Specific capacitance for both electrodes and volumetric capacitance of TTO hybrid structure electrode at different scan rates and f) different specific currents. g) The Nyquist plot of the oxidized Ta_4_C_3_T*_x_* MXene electrode and TTO hybrid structure electrode. The inset shows the electrical equivalent circuit. h) CV curve of the TTO hybrid structure electrode at 100 mV s^−1^. The pink and blue areas show the direct contributions of the capacitive and diffusion mechanisms respectively. i) Capacity contribution from capacitive and diffusion‐controlled kinetic processes at different scan rates for the TTO hybrid structure electrodes.

The electrochemical properties of the TTO hybrid structure were characterized by two‐electrode system. The TTO hybrid structure and oxidized Ta_4_C_3_T*_x_* MXene based electrodes were fabricated using a 8:1:2 weight ratio of MXene material, Super P carbon black and polyvinylidene fluoride (PVDF). The cyclic voltammetry (CV) and galvanostatic charge/discharge (GCD) were measured in the presence of a polyvinyl alcohol/phosphoric acid (PVA/H_3_PO_4_) solid electrolyte. The CV profiles of the oxidized Ta_4_C_3_T*_x_* MXene electrode and the TTO hybrid structure electrode were quasi‐rectangular at scan rates ranging from 1 to 100 mV s^−1^ with near mirror symmetry among all CV profiles, indicating that the majority of capacitance is associated with the electric double layer capacitance (EDLC) mechanism (**Figure** **4**a,b). Additionally, GCD curves of the oxidized Ta_4_C_3_T*_x_* MXene electrode and the TTO hybrid structure electrode feature nearly triangular shapes with extremely low internal resistance at the beginning of the discharge curve (Figure 4c,d). This reflects the pseudocapacitive nature of the oxidized MXene and TTO hybrid structure electrodes.^[^ ^40^ ^]^ In particular, the TTO hybrid structure exhibited significantly greater capacitance when compared with the oxidized Ta_4_C_3_T*_x_* MXene material. The observed specific capacitance values for TTO were more than twofold higher than oxidized Ta_4_C_3_T*_x_* MXene material at the same scan rate or specific current (Figure 4e,f). In fact, the new TTO hybrid structure electrode showed higher volumetric capacitance than most of the recently reported biomaterials‐based electrodes (Table S2, Supporting Information).

The electrochemical impedance spectroscopy analysis was used to investigate the ion‐diffusion/transport resistance of the TTO electrode in the frequency range of 0.01 Hz to 200 kHz at open‐circuit‐potential measurements. The impedance spectra of the oxidized Ta_4_C_3_T*_x_* MXene electrode and the TTO electrode form a small arc and a spike at the higher and lower frequency regions respectively. The TTO hybrid structure electrode clearly showed lower electrolyte resistance than the oxidized Ta_4_C_3_T*_x_* MXene electrode (Figure 4g). This may be due to the higher concentration of tantalum oxide nanoparticles on the surface of the TTO. As a result, in the equivalent electrical circuit model of the TTO hybrid structure electrode, there is a Warburg impedance element (W) representing linear diffusion under semi‐infinite conditions. Furthermore, the solution resistance (*R*_s_) signifies the electrolyte resistance and a constant phase‐element (*C*_dl_) obtained by EDLC of the TTO (Figure 4g and inset).

The electrochemical mechanism measurements of TTO hybrid structure were further investigated based on the following equation:

(1) $$\begin{array}{c} i\;\;=\;\;k_{1}\nu +k_{2}\nu^{1/2} \end{array}$$

This equation includes two scan rate‐related terms at a fixed scan rate and potential. The *k*_1_ν term is attributed to the current density contributed by the fast‐kinetics process in both electric double‐layer capacitance and Faraday pseudocapacitance.^[^ ^41^ ^]^ The *k*_2_ *v* ^1/2^ term is related to the current density contributed by a slow diffusion‐controlled process.^[^ ^41^ ^]^ Both constants, *k*_1_ and *k*_2_ are obtained from a different form of the above equation in a log‐log plot, as shown below.

(2) $$\begin{array}{c} i/\nu^{1/2}=\;\;k_{1}\nu^{1/2}+k_{2} \end{array}$$

The contribution of the fast‐kinetics process for various sweep rates is shown in Figure 4h,i and Figure S10, Supporting Information. The decoupling result for the TTO hybrid structure electrode at 100 mV s^−1^ offers a remarkable fast‐kinetics contribution of 87.0% (Figure 4h). Furthermore, while the fast capacitance remained unaffected by scan rate, the contribution of slow capacitance increases at lower scan rates (Figure 4i). This effect is attributable to the oxygen‐containing terminal groups on the surface of the TTO hybrid structure, which facilitates electrochemical Faradaic reactions to result in a greater contribution of diffusion‐controlled mechanisms at these lower scan rates.

These findings were confirmed with measurements from a three‐electrode system using the same electrolyte against an Ag/AgCl reference electrode. The results further elucidated the pseudocapacitance effect of Ta_2_O_5_ and other oxygen‐containing functional groups in acidic electrolytes. The TTO hybrid structure electrode exhibited EDLC behavior with quasi‐pseudocapacitance behavior (Figure S11a, Supporting Information). The oxidized surface of TTO may significantly increase the wettability and enlarge the ion‐accessible surface area, facilitating rapid ion diffusion/transportation into the internal pores. The possible redox reaction of Ta_4_C_3_T*_x_* MXene in an acidic medium is depicted in Equation (3).^[^ ^42^ ^]^

(3) $$\begin{array}{c} {\text{Ta}}_{4}C_{3}O_{x}{\left(\text{OH}\right)}_{y}+\;\delta H^{+}+\;\delta e^{-}↔{\text{Ta}}_{4}C_{3}O_{x-\delta }{\left(\text{OH}\right)}_{y-\delta } \end{array}$$

The possible redox reaction of Ta_2_O_5_ on Ta_4_C_3_T*_x_* MXene in an acidic medium is shown in Equation (4).^[^ ^43^ ^]^

(4) $$\begin{array}{c} {\text{Ta}}_{2}O_{5}+\;\beta H^{+}+\;\beta e^{-}↔H_{\beta }{\text{Ta}}_{2}O_{5} \end{array}$$

The normalized capacitance value for the two‐electrode system included contributions of two TTO hybrid structure electrodes, while the three‐electrode system used a single TTO hybrid structure electrode. Therefore, the values derived from three‐electrode measurements should be approximately two times of the values obtained from two‐electrode measurements (Figure 4e; Figure S11b, Supporting Information).^[^ ^38^ ^]^ Thus, the obtained results from the three‐electrode and two‐electrode systems of the TTO hybrid structure are in agreement with each other.

### 2.5 Ragone Plot of Ta_4_C_3_T*_x_* MXene‐Tantalum Oxide Electrode Supercapacitor

Next, we wanted to evaluate and compare the performance of the TTO hybrid structure electrode with literature‐reported organic/inorganic energy storage materials for bio‐implantable applications. Ideally, the best bioelectrode materials for implantable supercapacitors should possess excellent energy and power density in a single product, while having low individual component toxicity in case of damage and uncontrolled failure. In particular, currently used lithium‐ion batteries and their toxic electrolytes in cardiac pacemakers, neurostimulators, cochlear implants, and spinal cord stimulators have been reported to seriously jeopardize patient safety in cases of premature failure.^[^ ^44^, ^45^ ^]^

**Figure 5:**
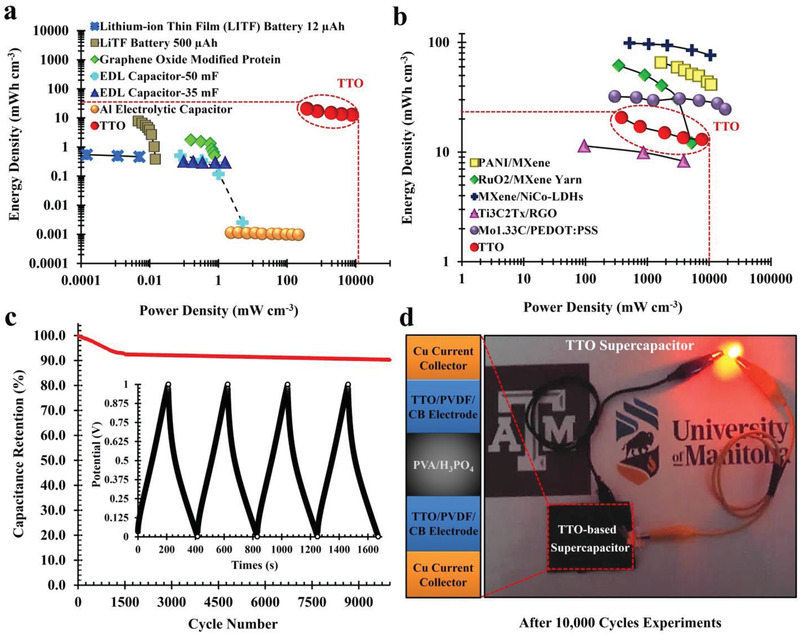
Comparison of TTO supercapacitor with some of the previously reported organic and inorganic electrode materials. a) Ragone plots comparing the performance of TTO with electrical double layer (EDL) capacitors (35 and 50 mF, 300 µF/3 V), graphene oxide modified protein electrode supercapacitor, aluminum electrolytic capacitor (12 µA h/3.3 V) and lithium‐ion thin film battery (LiTF, 500 µA h/5 V).^[^ ^45^, ^46^, ^47^, ^48^, ^49^, ^50^, ^51^, ^52^ ^]^ Energy density/power density of the TTO is significantly higher than above‐mentioned electrodes. b) Ragone plots comparing energy and power densities of the TTO hybrid structure supercapacitor to PANI/Ti_3_C_2_T*_x_*_,_^[^ ^42^ ^]^ RuO_2_/MXene yarn,^[^ ^43^ ^]^ Mo_1.33_C/PEDOT:PSS,^[^ ^43^ ^]^ Ti_3_C_2_Tx/RGO,^[^ ^53^ ^]^ and MXene/NiCo‐LDHs.^[^ ^54^ ^]^ c) Supercapacitor cycling stability, volumetric capacitance retention, and charge–discharge cycle at a current density of 1 A g^−1^. d) The image shows an LED powered by the TTO supercapacitor electrodes. Zoom‐view panel shows the schematic view of TTO‐based solid‐state supercapacitor containing PVA/H_3_PO_4_ gel electrolyte. The picture demonstrates that TTO supercapacitor was able to successfully power the LED.

In the current study, Ragone plots are presented to compare the volumetric performance of the TTO hybrid structure supercapacitor with several previously‐reported biocompatible supercapacitors and MXene‐based electrode supercapacitors (**Figure** **5**a,b). The TTO hybrid structure electrodes were packaged into a symmetric supercapacitor using copper current collectors and a PVA/H_3_PO_4_ solid electrolyte. Our data confirm that the TTO hybrid structure supercapacitor was superior to almost all other bioelectrode materials, including EDL capacitors, biophilized graphene oxide modified protein electrode supercapacitor, aluminum electrolytic capacitors, and lithium‐ion thin film batteries (Figure 5a and Table S2, Supporting Information).^[^ ^46^ ^]^ Additionally, the fluorine‐free TTO supercapacitor has competitive energy and power densities with other previously published MXene‐based supercapacitors (Figure 5b). Importantly, its performance also exceeds that of many currently reported non‐MXene carbon‐based electrodes, including graphene/CNT nanocomposites (165 F cm^−3^),^[^ ^47^ ^]^ graphene‐based electrodes (260 F cm^−3^),^[^ ^48^ ^]^ activated carbons (60–100 F cm^−3^),^[^ ^49^, ^50^ ^]^ and carbide‐derived carbons (180 F cm^−3^).^[^ ^51^, ^52^ ^]^ Finally, the TTO electrode possesses excellent areal efficiency when compared with other recently reported supercapacitor electrode materials (Figure S12, Supporting Information).

In addition to its excellent capacitance properties, the TTO displayed excellent long‐term stability over 10 000 cycles with capacitance retention maintained over 90% of the initial performance (Figure 5c). The capacitance retention was stabilized after 1500 cycles, with only an additional 2.4% decrease in capacitance over the subsequent 8500 cycles. Together, these data confirm that the TTO hybrid structure supercapacitor synthesized in the current study possesses long cycle stability, excellent volumetric capacitance and high charge/discharge rate performance. As a proof‐of‐concept, the energy storage performance of the TTO hybrid structure supercapacitor was functionally assessed by connecting it to a light‐emitting diode (LED); the experiment demonstrated that the symmetric TTO was able to successfully power the LED (Figure 5d).

### 2.6 Biocompatibility of Ta_4_C_3_T*_x_* MXene‐Tantalum Oxide

**Figure 6:**
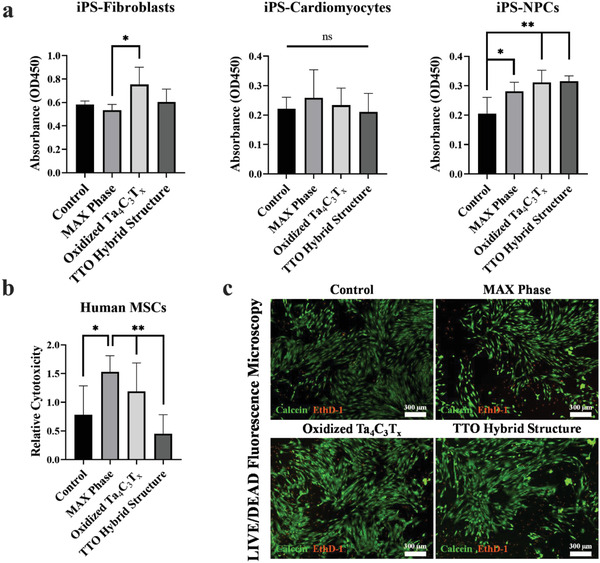
Assessment of biocompatibility of the Ta_4_AlC_3_ MAX phase and Ta_4_C_3_T*_x_* MXene‐tantalum oxides materials with human cells. a) The MAX phase, oxidized Ta_4_C_3_T*_x_*, and TTO materials were cocultured with human iPSC‐derived‐ fibroblasts, cardiomyocytes, and neural progenitor cells (NPCs) for 24 h. WST‐1 proliferation assay was performed to evaluate cytocompatibility of materials. Our data demonstrate that MXene was compatible with all three cell types, as coculture with biomaterial did not affect cellular proliferation compared to control group. b) Cytotoxicity evaluation of the MAX phase, oxidized Ta_4_C_3_T*_x_*, and TTO hybrid structure was assessed by LDH release after coculturing with human MSC for 24 h. LDH data show no significant difference among different MXene groups and the control group. c) LIVE/DEAD assay was performed using the fluorescent dye to assess biocompatibility of human MSC with the material. After coculture with different forms of MXene, MSC were stained with Calcein (for live cells, green) and EthD‐1 (for dead cells, red). Images were captured using Nikon Ti‐2 fluorescent microscope. No significant difference in viability between different groups was detected. (*n*=3–4 per group). (“ns” = statistically no significant difference, * = *p* < 0.05 and ** = *p* < 0.01).

We also investigated the biocompatibility of oxidized TTO electrode with human induced pluripotent stem cells (hiPSC)‐derived cardiomyocytes, neural progenitor cells (NPCs), and fibroblasts. The hiPSC‐derived cells were obtained using our established differentiation protocols (Figure S13, Supporting Information).^[^ ^55^ ^]^ When MXene‐based materials (at a concentration 50 µg mL^−1^) were cocultured with these cells for 24 h, assessment of cytotoxicity using the WST‐1 assay showed that all forms of the Ta_4_C_3_T*_x_* MXenes were compatible with cardiomyocytes, NPCs, and fibroblasts (**Figure** **6**a).

Additionally, TTO‐based bio‐electrodes may also be beneficial in the development and post‐delivery monitoring of functional cell‐based tissue constructs. Bone marrow‐derived mesenchymal stem cells (MSC), a commonly used cell type in tissue engineering, were found to be biocompatible with all forms of Ta_4_C_3_T*_x_* MXene‐based samples used in this study. When cocultured with materials for 72 h, assessment of cytotoxicity by LDH assay showed excellent residual viability of cells in all groups as there were no significant differences in cytotoxicity between different MXene groups and the control group (Figure 6b). On the other hand, MAX phase, due to the presence of Al, demonstrated lower biocompatibility to the cells. Representative images captured using a fluorescence‐based live/dead assay also confirmed this finding (Figure 6c). These data confirm that oxidized TTO electrode is biocompatible and it can be used for implantable bioelectronic devices and tissue engineering applications.

## 3 Conclusion

This study reported the first fluorine‐free synthesis and application of Ta_4_C_3_T*_x_* MXene‐tantalum oxides hybrid structure material for energy storage applications. The TTO‐based electrode showed excellent volumetric capacitance compared to previously reported biocompatible electrodes. Furthermore, the TTO hybrid structure is highly biocompatible with different types of human cells, which is highly beneficial for future applications in bioelectronics and biosensors. Finally, when assembled into a symmetric supercapacitor, the TTO hybrid structure material possessed high energy/power densities and long‐term cyclability. The stability of TTO electrodes was estimated to be over 10 000 cycles.

## 4 Experimental Section

### Fluorine‐Free Synthesis of Oxidized Ta_4_C_3_T*_x_* and Ta_4_C_3_T*_x_* MXene‐Tantalum Oxide Hybrid Structure

Ta_4_C_3_T*_x_* MXene nanosheets were partially exfoliated using hydrochloric acid (HCl). To do so, Ta_4_AlC_3_ MAX Phase powder was incubated in 6 m solution of HCl in water at 37 °C for 72 h in a shaking incubator at 260 rpm. The precipitates were collected after washing with ultrapure distilled water by spinning at 5000 rpm for 5 min each. The precipitates were freeze dried for 48 h and subsequently air dried at 60 °C. The complete etching, exfoliation, and surface modification of the obtained material (dry powder) was achieved by treating it with potassium hydroxide (KOH, 6 m) at room temperature for 90 h. The edge exfoliation of specimens was obtained by centrifugation at 5000 rpm followed by several washing steps and vacuum lyophilization (−80 and −54 °C) for 48 h to avoid uncontrolled oxidization. The powder was then double‐dried in an atmospheric oven at 50 °C for 48 h. The resultant nanocomposite obtained at this step was labeled as oxidized Ta_4_C_3_T*_x_* at room temperature (22 °C). For further functionalization and oxidation, the treated Ta_4_C_3_T*_x_* nanosheets were subjected to thermal treatment at 220 °C for 2 h under moderate air heating and labeled as TTO hybrid structure (220 °C).

### Physicochemical Characterization

The structural properties of materials were characterized using an FEI Nova NanoSEM 450 (Thermo Fisher Scientific), FEI Talos F200X S/TEM (Thermo Fisher Scientific), Thermo Nicolet Nexus 870, and Kratos Axis Ultra XPS at the Manitoba Institute of Materials (MIM), University of Manitoba, Winnipeg, Canada. The SEM samples were mounted on pin stubs using carbon tape and coated with a gold‐palladium (Au‐Pd) coating to enable high magnifications. XRD peaks of powdered samples were collected in the range from 5 to 80° 2‐theta using continuous scan mode with a scan rate of 3° min^−1^ and report interval of 0.05°. The measurement of specific surface area of the materials was determined by the BET analysis.

### Electrode Fabrication, Electrical, and Electrochemical Measurements

The TTO electrodes were fabricated using the following procedures. Each TTO hybrid structure electrode was synthesized using 8:1:2 ratio of TTO hybrid structure (160 mg), Super P carbon black (20 mg), and PVDF (40 mg) in *N*‐methyl‐2‐pyrrolidone solvent. The slurry prepared by mixing these components was brushed on a carbon paper and pressed after drying in a vacuum oven at 70 °C for 24 h. The capacitance properties of the prepared TTO electrodes were characterized by using two‐ and three electrode systems at room temperature.

Cyclic voltammetry and the constant current charge–discharge measurements were performed on Autolab electrochemical workstation (PGSTAT302 N model) and CH Instrument 640E Bipotentiostat. The specific capacitance for cyclic voltammetry‐based measurement was calculated according to the following equation:

(5) $$\begin{array}{c} C\;\;=\;\;\frac{1}{2v\;m\;\Delta V\;}\;\int IdV \end{array}$$

where *C*, *I*, ν, *m*, and Δ*V* are the specific capacitance, current, scan rate (V s^−1^), weight of electrode, and scanning potential window, respectively. The constant current charge–discharge test was performed for specific capacitance. Values were calculated using the following equation:

(6) $$\begin{array}{c} C\;\;=\;\;\frac{I\;\Delta t}{m\;\Delta V} \end{array}$$

where *I*, Δ*t*, and Δ*V* are respectively discharge current, discharge time, and discharge potential window.^[^ ^38^ ^]^ The energy density and power density of the device were calculated as *E = C*(Δ*V*)^2^/7.2 and *P = 3600 E*/Δ*t*, where *E* and *P*, are energy and power densities, respectively. Using the density of the packed electrolyte (2.3 × 10^−3^ kg cm^−3^), the volumetric energy density (*E*_vol_), and volumetric power density (*P*_vol_) were calculated using the following equations:

(7) $$\begin{array}{c} E_{\text{vol}}\;\left(Wh\;cm^{-3}\right)=\;E\;\;\times \;\;\rho \end{array}$$

(8) $$\begin{array}{c} P_{\text{vol}}\;\;=\;\;3600\;\;\times \;\;\frac{E_{\text{vol}}}{\Delta t} \end{array}$$

The CV and GCD measurements were performed with all solid‐state two‐electrode system in the presence of PVA and H_3_PO_4_ gel electrolyte. For this, a gel electrolyte solution was prepared by mixing 10 g of PVA and 10 g of H_3_PO_4_ in 100 mL of deionized water at 85 °C. The solution was incubated in an oven at 40 °C for a week to solidify. A thin layer of the PVA/H_3_PO_4_ electrolyte was sandwiched between two active electrodes of the same size and mass and subjected to a hot‐pressing step at 10.9 psi. The copper (Cu) foils were attached to the other side of the TTO electrode to be used as the current collector. For Ragone plots the total mass of the packaged TTO‐based supercapacitor (959 mg) was used, including TTO hybrid structure electrodes, electrolyte, carbon blacks, and PVDF to calculate and evaluate the total energy or power densities.

The capacitance of TTO‐based electrodes was further characterized by using a three‐electrode system at room temperature with phosphoric acid (H_3_PO_4_) solution as electrolyte. The platinum (Pt) and silver/silver chloride (Ag/AgCl) were used in the experiment as the counter electrode and the reference electrode, respectively.

### Density Measurement

The gravimetric capacitance of the TTO electrode is converted to volumetric capacitance by Archimedes’ Principle. The density of TTO electrode was calculated using the following equation:

(9) $$\begin{array}{c} \rho \;\;=\;\;\frac{W_{a}}{W_{\text{ax}}}\rho_{\text{ax}} \end{array}$$

where *W*_a_, *W*_ax_, ρ, and ρ_ax_ are the weight of the sample in air, weight of the sample in the auxiliary liquid of known density, the density of the sample and auxiliary liquid, respectively.

### Assessment of Energy Storage Performance of Ta_4_C_3_T*_x_* MXene‐Tantalum Oxide Supercapacitor

The energy storage performance of TTO was assessed using a light‐emitting diode (LED). The TTO electrode in the supercapacitor was charged and was connected in an LED output and the performance was observed.

### Induced Pluripotent Stem Cells Generation, Culture, and Differentiation

hiPSCs were generated from peripheral blood mononuclear cells (PBMC) isolated from human blood (collected from healthy individuals).^[^ ^21^ ^]^ All protocols were approved by the University of Manitoba Health Research Ethics Board (B2015:025, HS18974). To reprogram PBMCs toward iPSCs a commercial reprogramming kit (CytoTune‐iPS 2.0 Sendai Reprogramming Kit) was used (A16517, ThermoFisher Scientific, US). The detailed procedure is described in previously published studies.^[^ ^56^, ^57^ ^]^

The hiPSCs were cultured in TeSR‐E8 (0 5990, STEMCELL Technologies) on Geltrex (A1413302, Gibco) and allowed to differentiate toward fibroblast, cardiomyocytes (CMs) and NPCs using our previously published protocols.^[^ ^55^ ^]^ Briefly, embryoid bodies (EBs) were prepared in suspension in low attachment plates (174 932, Thermo Scientific) and plated onto gelatin‐coated plates on Day 8 (PMEF‐CFL‐P1, EMD Millipore). They were allowed to differentiate spontaneously toward fibroblasts, which were manually dissected from the culture plate and characterized by staining for HSP47 (ab77609, Abcam) and FSP (ab11333, Abcam) (Figure S13, Supporting Information). The cell populations were enriched over several passages to ensure a pure fibroblast population.

The hiPSCs were differentiated to cardiomyocytes using following protocol: iPSCs (>passage 20) were passaged onto Geltrex‐coated plates using Versene Solution (15 040 066, Gibco) and grown till the cells reached ≈85% confluency. The medium was replaced with CDM3, consisting of RPMI 1640 (61 870 036, Gibco) supplemented with 500 µg mL^−1^ recombinant human albumin (A9731, Sigma‐Aldrich), and 213 µg mL^−1^ L‐ascorbic acid 2‐phosphate (A8960, Sigma‐Aldrich). The culture medium was replaced on alternate days (48 h). At days 0–2, the medium was supplemented with 6 µM of the glycogen synthase kinase 3‐β inhibitor CHIR99021 (SML1046, Sigma‐Aldrich). On day 2, the medium was changed to CDM3 supplemented with 2 µM of the Wnt inhibitor‐ Wnt‐C59 (5.00496.0001, CalBiochem). Day 4 onward, the cells were cultured in medium without the inhibitors. The beating cells were observed from day 7. At day 10, medium was replaced with RPMI 1640 without glucose (11 879 020, Gibco), 500 µg mL^−1^ recombinant human albumin, and 213 µg mL^−1^ L‐ascorbic acid 2‐phosphate supplemented with 4 mM L‐lactic acid (71 720, Sigma‐Aldrich) for metabolic enrichment of cardiomyocytes. The cardiomyocytes were characterized by immunofluorescence staining for sarcomeric alpha actin (ab9465, Abcam) and MYH6 (ab50967, Abcam).

The differentiation of iPSCs toward NPCs was carried out using EB method by initiating the treatment with the TGF‐beta/Smad inhibitor SB 431 542 (16‐141, Torcis) for 2 days. The EBs were then plated on polyornithine‐coated plates on day 5 (A004, Merck Millipore). The neural rosettes were visually identified at day 7–10. After that, the rosettes were excised and grown on polyornithine‐coated plates in STEMPRO NSC SFM kit (A1050901, Gibco). The NPC characterization was carried out by immunostaining for NESTIN (sc‐23927, Santa Cruz Biotechnology) and PAX6 (sc‐81649, Santa Cruz Biotechnology).

### Human Mesenchymal Stem Cells Culture

Human bone marrow derived MSC were purchased from Lonza (PT 2501, CA10064‐080) and cultured in low‐glucose DMEM (10 567 014, Gibco) according to previously published protocols.^[^ ^58^ ^]^

### Cell Proliferation Assay

Human iPSC‐derived fibroblasts, cardiomyocytes, and NPCs were plated on 96‐well plates and cocultured with or without the raw MAX phase and oxidized TTO composites at a concentration of 50 µg mL^−1^ for 24 h. Then, the cell proliferation was assessed using the WST‐1 proliferation kit (K301, BioVision).

### Assessment of Cytotoxicity

To assess cytotoxicity, human MSC were cultured with different forms of MXene for 24 h at a concentration of 50 µg mL^−1^. To evaluate cytotoxicity, LDH release from damaged cells (if any) was measured in the supernatant using a Cytotoxicity Detection Kit (MK401, Takara Bio).

### Assessment of Cellular Viability using LIVE/DEAD Assay

To assess the effect of TTO MXene on cell viability, LIVE/DEAD assay was performed. Briefly, human MSC (2 × 10^5^) were plated on 96‐well plates and cocultured with or without the MAX phase and TTO composites for 72 h. The cells were then stained using a LIVE/DEAD Viability/Cytotoxicity Kit (L3224, Invitrogen) for 30 min and then visualized using Nikon Eclipse Ti‐2 fluorescence microscope. Calcein was detected using the GFP Filter (Ex480/Em535) and EthD‐1 was detected using the TRITC Filter (Ex540/Em605).

### Statistical Analysis

Data were reported as mean ± SD unless otherwise specified. Comparison of data between multiple groups was performed using one‐way analysis of variance (ANOVA) followed by Tukey's post‐hoc multiple comparison test, and analysis between two groups was made using an unpaired Student's *t*‐test (two‐tailed). Statistical analysis was performed using GraphPad Prism 8.0.1 (San Diego, USA). Statistical significance was defined as *p* < 0.05.

## Conflict of Interest

The authors declare no conflict of interest.

## Author Contributions

The study was conceptualized and designed by A.R., A.A., and S.D. A.R., A.A., G.L.S., W.Y., and Y.C. carried out the experiments and acquired the data. A.R., A.A., W.Y., A.A.P., and S.D. interpreted the data and performed statistical analysis. A.R., A.A., W.Y., and S.D. designed the figures. A.R., A.A., and S.D. drafted the manuscript. All authors have read and approved the final manuscript.

## Supporting information

Supporting Information

## Data Availability

The data that support the findings of this study are available from the corresponding author upon reasonable request.
