# Supplementary material for: Development of Fluorine‐Free Tantalum Carbide MXene Hybrid Structure as a Biocompatible Material for Supercapacitor Electrodes
Source: Adv Funct Mater. 2021 May 24;31(30):2100015. doi: 10.1002/adfm.202100015 (PMC8889894; doi:10.1002/adfm.202100015)
Supplement: Supplementary file 1 — Supporting Information [file ADFM-31-0-s001.pdf]

© 2021 Wiley-VCH GmbH

ADVANCED FUNCTIONAL MATERIALS

Supporting Information

for *Adv. Funct. Mater.,* DOI: 10.1002/adfm.202100015

Development of Fluorine-Free Tantalum Carbide MXene Hybrid Structure as a Biocompatible Material for Supercapacitor Electrodes

*Alireza Rafieerad, Ahmad Amiri, Glen Lester Sequiera, Weiang Yan, Yijun Chen, Andreas A. Polycarpou, and Sanjiv Dhingra**

Supplementary Information

Development of Fluorine-Free Tantalum Carbide MXene Hybrid Structure as a Biocompatible Material for Supercapacitor Electrodes

Alireza Rafieerad[a], Ahmad Amiri[b], Glen Lester Sequiera[a], Weiang Yan[a], Yijun Chen[c], Andreas A. Polycarpou[b], Sanjiv Dhingra[a,*]

[a] *Regenerative Medicine Program, Institute of Cardiovascular Sciences, St. Boniface Hospital Research Centre, Department of Physiology and Pathophysiology, Rady Faculty of Health Sciences, University of Manitoba, Winnipeg, Canada*

[b] *J. Mike Walker '66 Mechanical Engineering Department, Texas A&M University, College Station, Texas 77843, United States*

[c] *Department of Aerospace Engineering, Texas A&M University, College Station, Texas, 77843, United States*

Keywords: Fluorine-free $Ta_4C_3T_x$ MXene; TTO hybrid structure; Biocompatible electrode; Human stem cells; Supercapacitors.

Correspondence:
Sanjiv Dhingra, PhD, FAHA, FAPS
Associate Professor
Regenerative Medicine Program
Director: Canada Italy Tissue Engineering Program
Institute of Cardiovascular Sciences, St. Boniface Hospital Research Centre
R-3028-2, 351 Tache Avenue, Winnipeg, R2H2A6, Canada
Email: sdhingra@sbrc.ca

<u>Table S1:</u> The XPS chemical component analysis of the Ta_4AlC_3 MAX phase and TTO hybrid structure. Atomic percentages are presented for each of the identified species.

**Sample**	**Ta**	**Al**	**C**	**O**	**Ta-Oxide**	**$Ta_4C_3O_x$**	**$Ta_4C_3(OH)_x$**
Ta_4AlC_3 MAX Phase	32.44	5.5	29.04	33.02	14.4	41.7	30.8
TTO Hybrid Structure	39.89	0.92	18.94	40.25	24.8	27.6	31.6

Table S2: The properties of TTO hybrid structure electrode (fabricated in the current study) were compared to the previously reported organic/inorganic electrode materials in the literature.

Bioelectrode Materials	Energy Storage Performance (ESP)	Organic Electrolyte	Device Application	Cell Type Compatibility	Number Cycles	Retention (%)	Ref
Titanium dioxide (TiO_2) nanotube	1.42 mF cm^{-2} (0.01-1 V s^{-1}, 50-150 μA cm^{-2})	Phosphate buffer saline (PBS)	Implantable supercapacitor	NA	5000	94	[1]
Carbon nanotube fiber	10.4 F cm^{-3} (11.4-20.8 F g^{-1}, 20-500 mV s^{-1}, 0.3-2.7 A cm^{-3})	PBS, serum, blood	Implantable supercapacitor	Primary mouse embryonic fibroblast	10000	98.3	[2]
Multiwall carbon nanotube (MWNT)-yarn	0.7 mF cm^{-2} (10-100 mV s^{-1})	PBS	Implantable device	NA	NA	NA	[3]
MWNT fiber-ferritin	2.6 mF cm^{-2}	PBS	Implantable device	NA	NA	NA	[3]
MWNT fiber-poly(3,4-ethylenedioxythiophene)-poly(styrene sulfonate)	4.3 mF cm^{-2}	PBS	Implantable device	NA	NA	NA	[3]
MWNT fiber-ferritin-PEDOT-PSS	2.0 F cm^{-3} (24.1 F g^{-1}, 32.9 mF cm^{-2}, 10-100 mV s^{-1}, 0.1 A cm^{-2})	PBS	Implantable device	NA	1000	98	[3]
MWCNT-manganese dioxide (MnO_2)	20 F g^{-1} (0.2–1.0 V)	NA	Energy storage biodevice	Human-derived fibroblast, African green monkey kidney	1000	99	[4]
Graphene-protein	534 F cm^{-3} (2.5 A g^{-1}, 100 mV s^{-1}, 2.5 A g^{-1})	NA	Implantable energy storage	Mouse embryo fibroblasts, fibroblast-like monkey kidney	5000	98	[5]
Silver-polyaniline-pectin (Ag-PANI-PEC)	290 F g^{-1} (2.5 A g^{-1}, 100 mV s^{-1})	PBS, serum, blood, urine	Implantable energy storage devices	Rat myoblast	250	80	[6]
DNA hydrogel	28.5 ± 2.2 F g^{-1} (1 A g^{-1})	PBS, urine	Implantable energy storage	Mouse embryonic fibroblast	1000	95	[7]
TiO_2 tubular array	84 F cm^{-3} (3.75 A cm^{-3}, 100 mV s^{-1})	PBS	Implantable energy storage	Primary mouse embryonic fibroblast	2000	83	[8]
Graphene-polypyrrole (PPy) foam	89.60 mF cm^{-2} (0.6-3.3 mA cm^{-2}, 5-500 mV s^{-1})	NA	Hand patchable device	NA	10000	75	[9]
MWCNT-PPy	5.17 mF cm^{-2} (200 mV s^{-1}, 0.10 mA cm^{-2})	NA	Skin-attachable healthcare	NA	5000	80	[10]
MWCNT-manganese tetroxide (Mn_3O_4)	8.90 F cm^{-3} (0.05-1 V s^{-1}, 3.0 A cm^{-3})	NA	Knee and arm patchable device	NA	30000	93	[11]
Tungsten-iron-molybdenum-oxide (Wox-FeOx-MoOx)	1.60 mF cm^{-2} (300 mV s^{-1}, 1 V s^{-1}, 0.05-0.15 mA cm^{-2})	PBS	Fingernail for human healthcare	NA	6000	99	[12]
Nitrogen-doped carbon microflakes (N-CMF)	363 F g^{-1} (5-100 mV s^{-1}, 1.5- 30 A g^{-1})	NA	Biocompatible supercapacitor	NA	10000	92.4	[13]
N-doped wrinkled graphene nanosheets (N-GNSs)	523 F g^{-1} (5-100 mV s^{-1}, 1.5- 30 A g^{-1})	NA	Biocompatible supercapacitor	NA	10000	94.8	[13]
N-doped honeycomb carbon (N-HC)	428 F g^{-1} (5-100 mV s^{-1}, 1.5- 30 A g^{-1})	NA	Biocompatible supercapacitor	NA	10000	96	[13]
Ag-zinc (Zn)	1.48 mAh cm^{-2} (1.4 mA cm^{-2}, 2 mV s^{-1}, 14.5-20 mA cm^{-2})	NA	Painted skin-tattoo battery	NA	25	NA	[14]
Reduced graphene oxide (rGO)	6.84 mF cm^{-2} (0.1 mA cm^{-2}, 1000 mV s^{-1}, 1.5 mA cm^{-2})	NA	Tailorable device	NA	20000	94.3	[15]
**TTO hybrid structure (Material in the current study)**	**447 F cm^{-3} (194 F g^{-1}, 1 to 100 mV s^{-1})**		**Implantable supercapacitor**	**Human iPSC-fibroblast, cardiomyocyte, neural progenitor cells, mesenchymal stem cells**	**10000**	**92.65**	**NA**

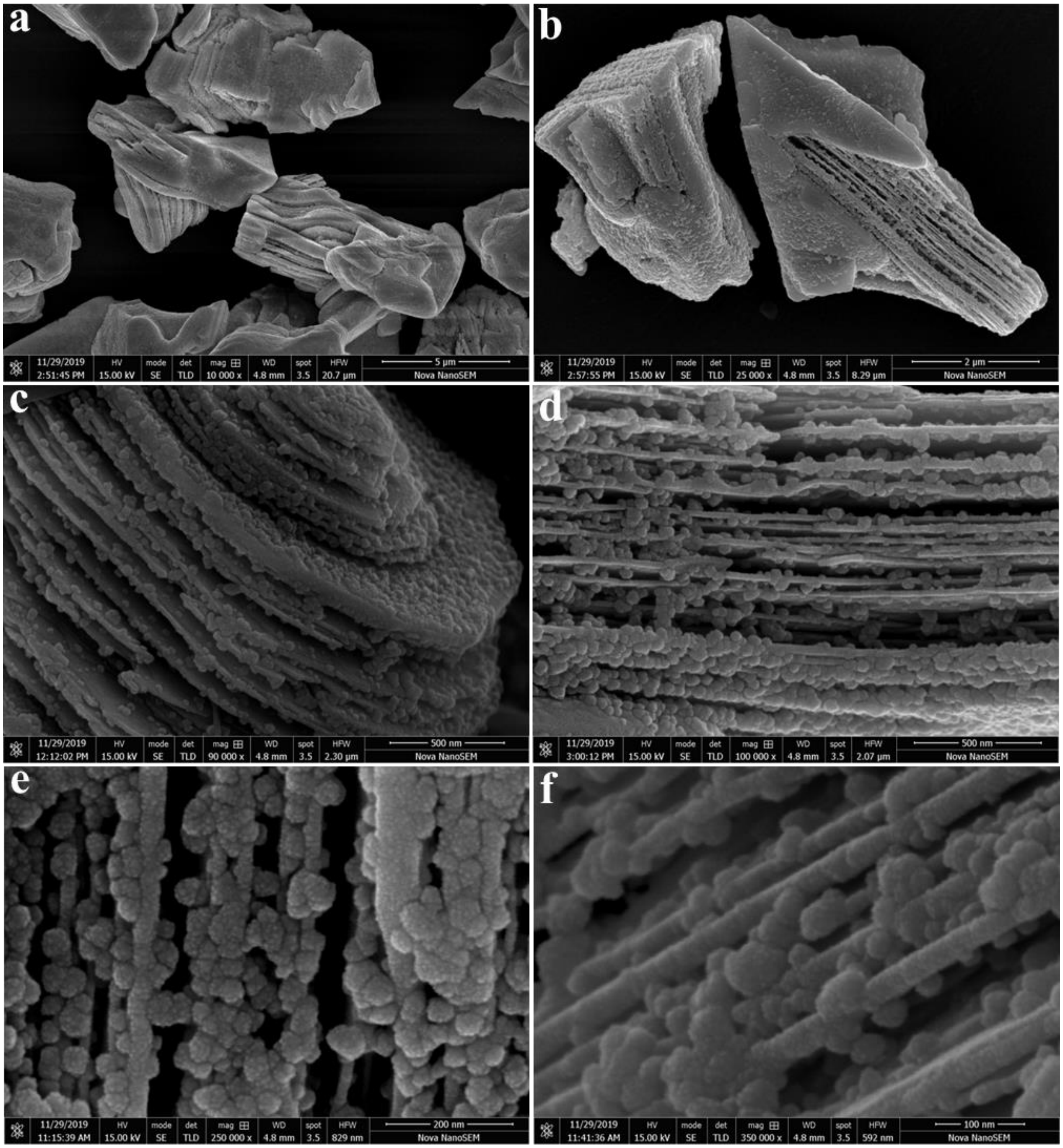

<u>Figure S1:</u> (a-f) High magnification FESEM images showing successful fluorine-free synthesis of TTO hybrid structure from the Ta_4AlC_3 MAX phase. The morphology of TTO nanostructure consists of exfoliated $Ta_4C_3T_x$ nanosheets decorated by tantalum oxide nanoparticles after thermal treatment at 220°C for 2 hours. The clusters of Ta-oxide particles on functionalized MXene nanosheets surface were grown during the synthesis process. The Ta-oxide nanoparticles were uniformly distributed in TTO structure.

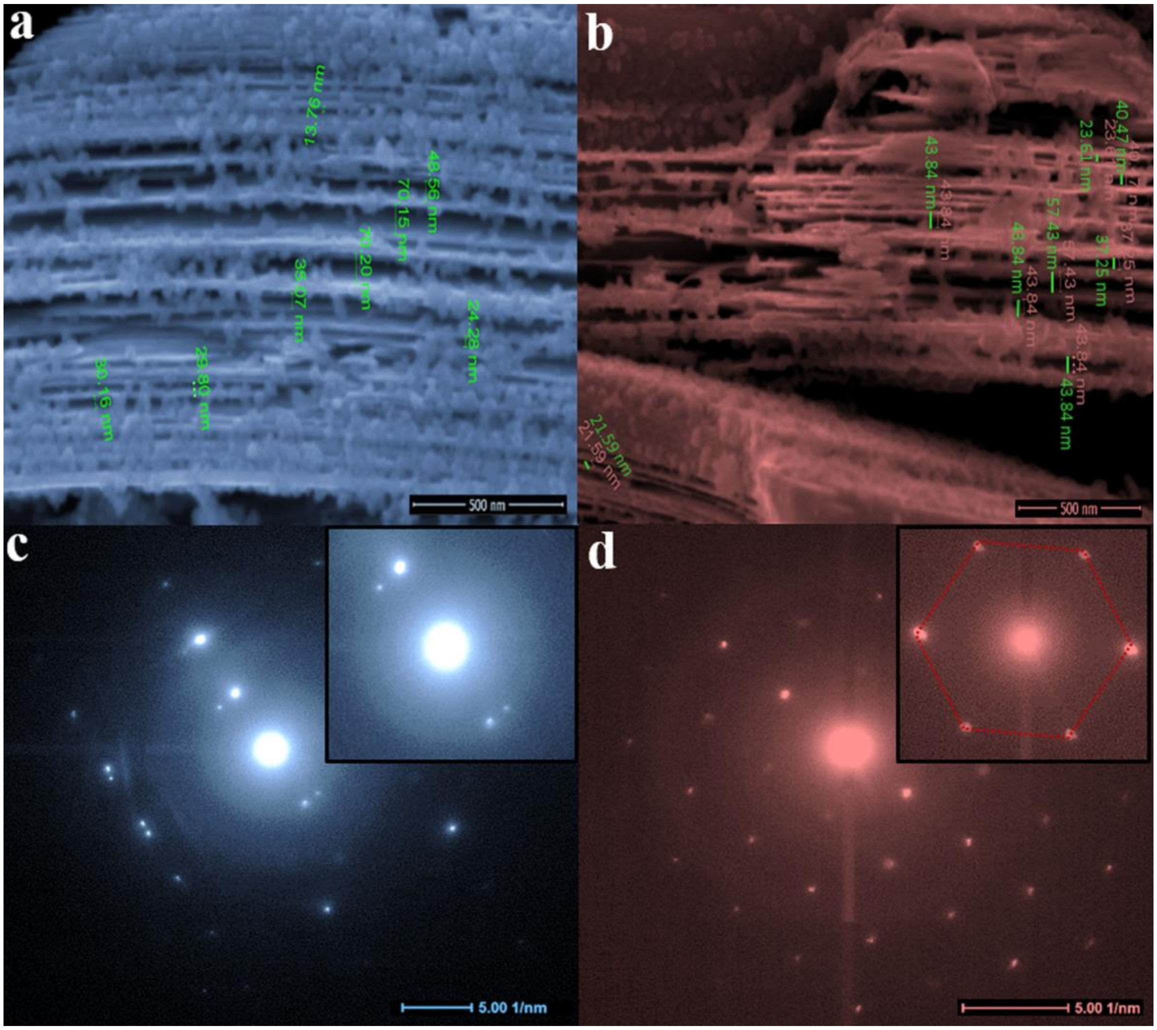

Figure S2: High magnification FESEM images showing the wall-to-wall interlayer space of surface-modified MXene nanosheets **(a)** before and **(b)** after thermal treatment at 220°C for 2 hours. The selected area-electron diffraction (SAED) patterns were compared to assess the crystallization of the **(c)** oxidized $Ta_4C_3T_x$ MXene and **(d)** TTO hybrid structures. The SAED patterns depicted a fully formed crystalline structure with well-defined hexagonal planes in TTO samples **(d**, **inset)**. Furthermore, TTO hybrid structure electrode material showed higher crystallization stability after the employed thermal treatment.

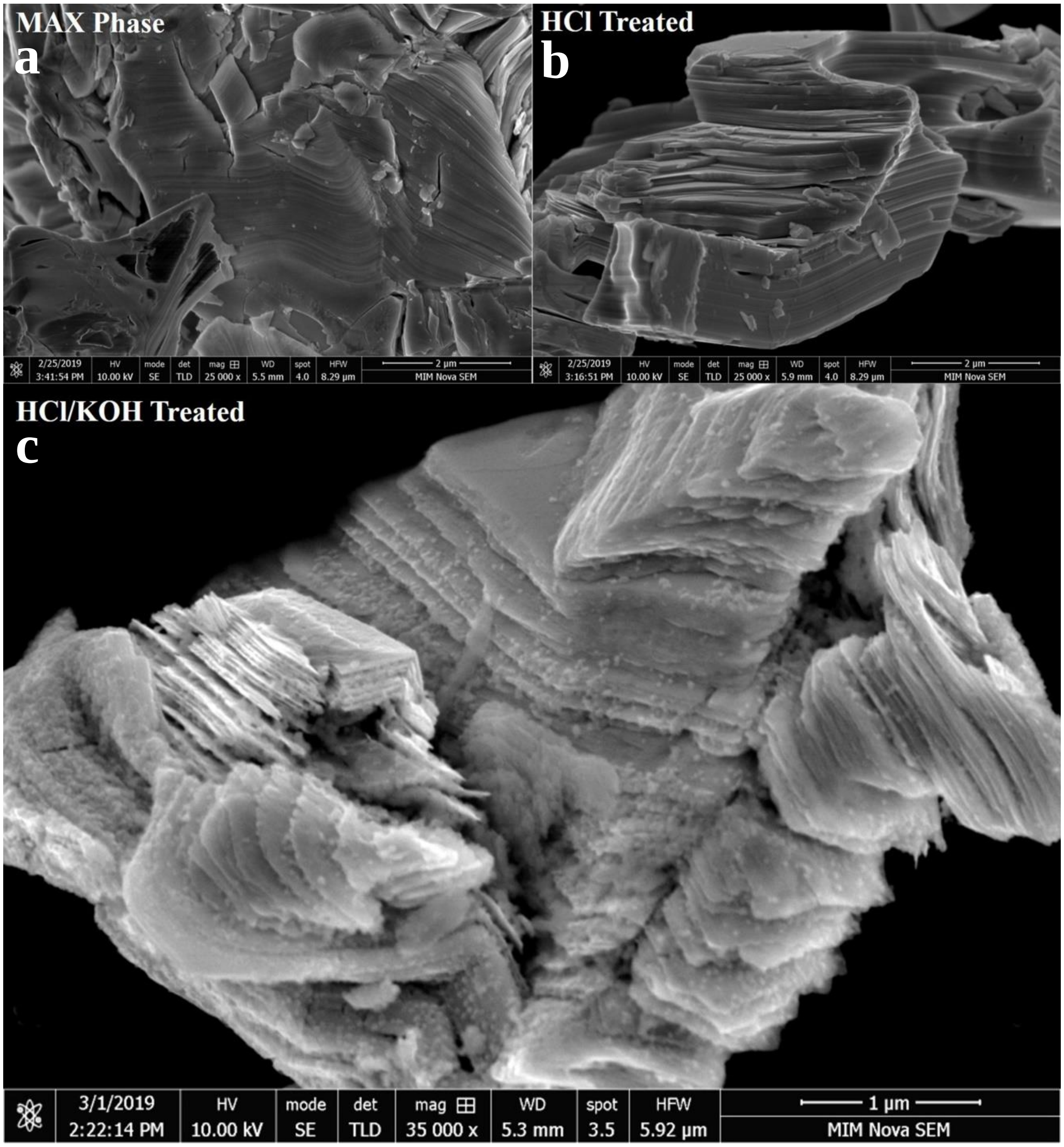

Figure S3: FESEM images showing the conversion of bulky Ta_4AlC_3 MAX phase to a TTO hybrid nanostructure. A fluorine-free HCl/KOH etching, exfoliation, and surface modification protocol was used to synthesize $Ta_4C_3T_x$ MXene nanosheets anchored with Ta-Oxide nanoparticles. Morphology illustration of the **(a)** bulky Ta_4AlC_3 MAX phase, **(b)** HCl-treated pre-MXene, and **(c)** HCl/KOH-treated TTO hybrid structures after thermal treatment at 220 °C. To overcome the blocked/slow kinetic reactions challenge in etching MAX phase in alkaline media, a hybrid two-step acid/alkaline etching method was utilized to synthesize and functionalize $Ta_4C_3T_x$ MXene nanosheets.

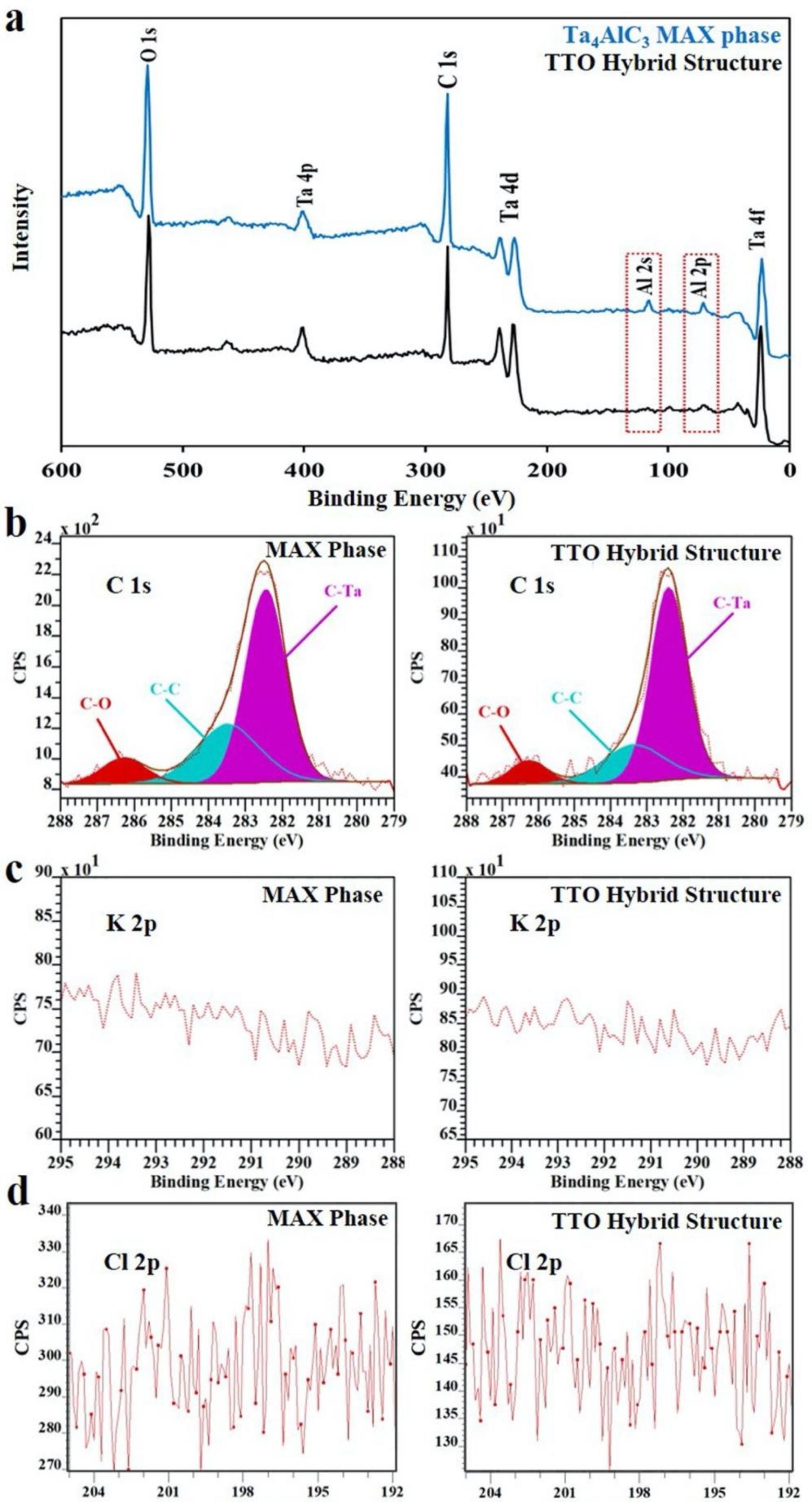
a
Ta_4AlC_3 MAX phase
TTO Hybrid Structure
O 1s
C 1s
Ta 4p
Ta 4d
Ta 4f
Al 2s
Al 2p
Intensity
Binding Energy (eV)
b
MAX Phase
TTO Hybrid Structure
C 1s
C-Ta
C-C
C-O
CPS
Binding Energy (eV)
c
MAX Phase
TTO Hybrid Structure
K 2p
CPS
Binding Energy (eV)
d
MAX Phase
TTO Hybrid Structure
Cl 2p
CPS
Binding Energy (eV)

<u>Figure S4:</u> XPS analysis of the raw Ta_4AlC_3 MAX phase and fluorine-free exfoliated oxidized TTO samples. **(a)** Wide survey of the MAX phase and TTO hybrid structure electrode material. The main peaks of Al 2p and Al 2s at the binding energy of (64-80 eV and 115-125 eV) were significantly decreased after thermal treatment of oxidized TTO heterostructure. This data confirms that the Al layer has been extracted from the structure of MAX phase material. The narrow scan Ta 4f, Ta 4p, C 1s and O 1a further confirmed that the oxidized TTO nanocomposite was successfully synthesized. **(b)** C 1s spectra of the MAX phase and TTO hybrid structure demonstrates that the counts-per-second intensity of C─C, C─O and C─Ta peaks were changed, and the peaks show different trend. **(c, d)** The fitting spectra of K 2p and Cl 2p also confirmed the complete removal of Cl and K of HCl and KOH from the TTO hybrid structure.

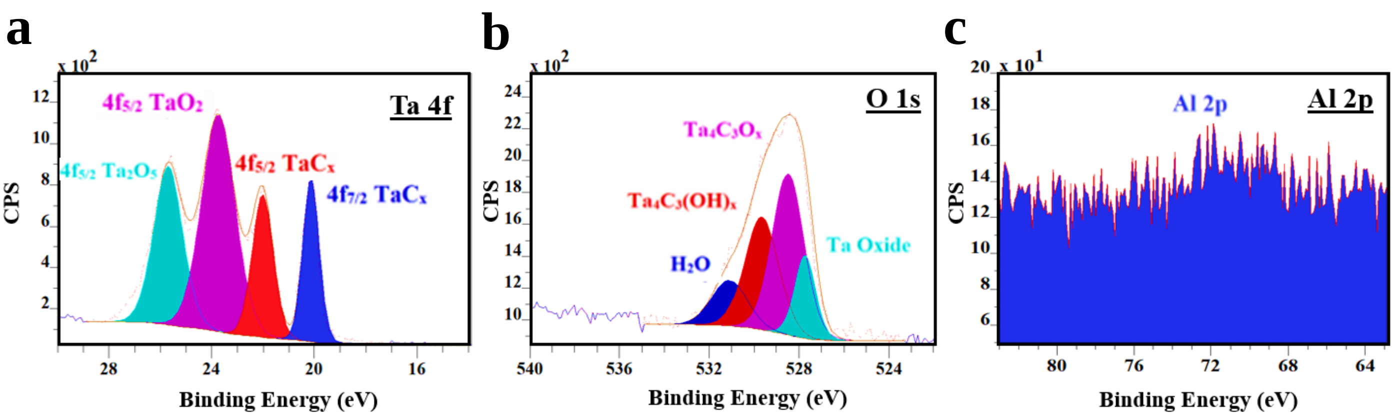

Figure S5: High-resolution narrow scan XPS spectra of the oxidized $Ta_4C_3T_x$ MXene material. **(a)** Ta 4f, **(b)** O 1s, and **(c)** Al 2p spectra showed the relative exfoliation and functionalization of Ta_4AlC_3 MAX phase prior to the thermal treatment. Briefly, the Ta 4f and O 1s spectra displayed the formation of MXene with OH surface functional groups along with Ta-oxides peaks. Also, the Al 2p narrow scan depicted the significant removal of Al from the structure of MAX phase.

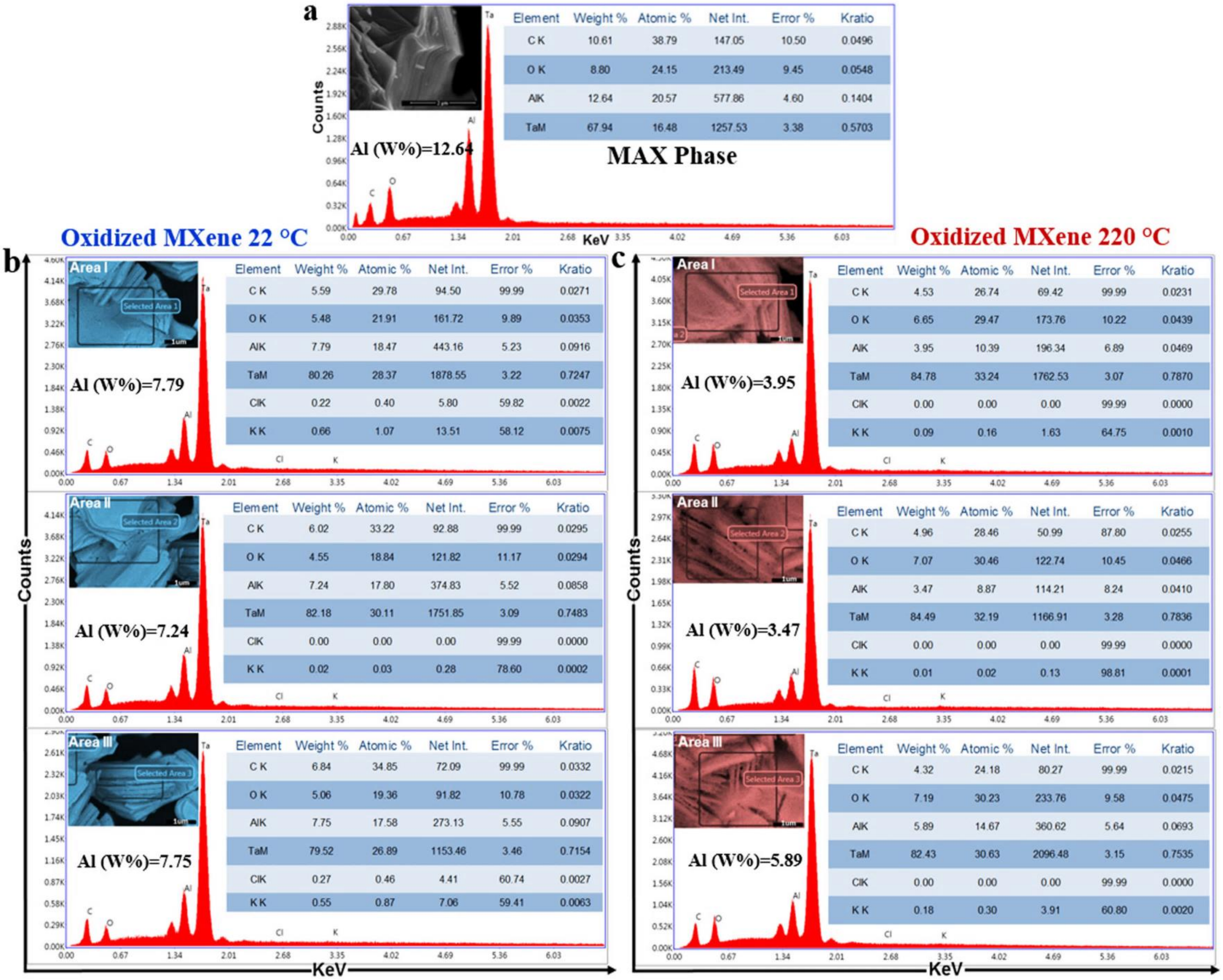

<u>Figure S6:</u> Microstructure and histogram elemental analysis of the **(a)** MAX phase, **(b)** oxidized $Ta_4C_3T_x$ MXene, and **(c)** TTO hybrid structure, after thermal treatment at 220°C for 2 hours. The EDS analysis was used to evaluate the elemental composition of the Ta_4AlC_3 MAX phase and TTO samples. The measured EDS difference between the oxygen, carbon, and tantalum weight percentage of MAX phase and TTO hybrid structure further confirmed the increase in oxidation. Our EDS results also confirmed the extraction of aluminum during the synthesis process. The EDS analysis demonstrated a significant elimination of Al from Ta_4AlC_3 MAX phase with a decrease in the weight percentage from 12.64% to 4.43% in TTO after thermal treatment.

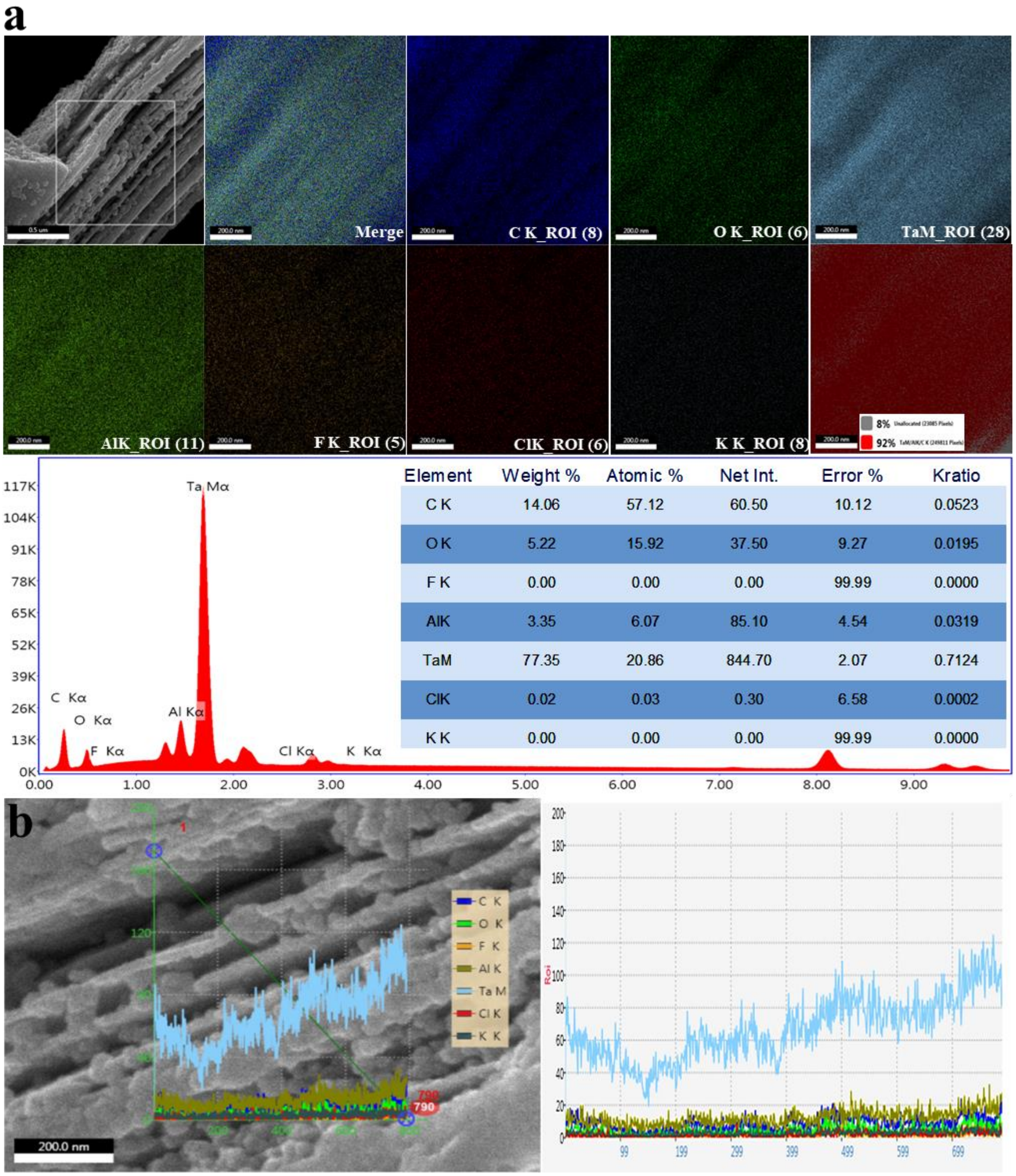

Element	Weight %	Atomic %	Net Int.	Error %	Kratio
C K	14.06	57.12	60.50	10.12	0.0523
O K	5.22	15.92	37.50	9.27	0.0195
F K	0.00	0.00	0.00	99.99	0.0000
AlK	3.35	6.07	85.10	4.54	0.0319
TaM	77.35	20.86	844.70	2.07	0.7124
ClK	0.02	0.03	0.30	6.58	0.0002
K K	0.00	0.00	0.00	99.99	0.0000

<u>Figure S7:</u> (a) EDS mapping and **(b)** line scan analysis further showing the elemental composition microstructure of TTO hybrid structure. This analysis depicts quantification of each element (C, O, Ta, Al, F, Cl, and K) in the structure of TTO nanocomposite. The EDS histogram confirmed absence of F, Cl, and K in the final structure of TTO. The EDS line scan analysis further confirmed the elemental composition of TTO hybrid structure. The line scan analysis also confirmed the composition of the TTO hybrid structure.

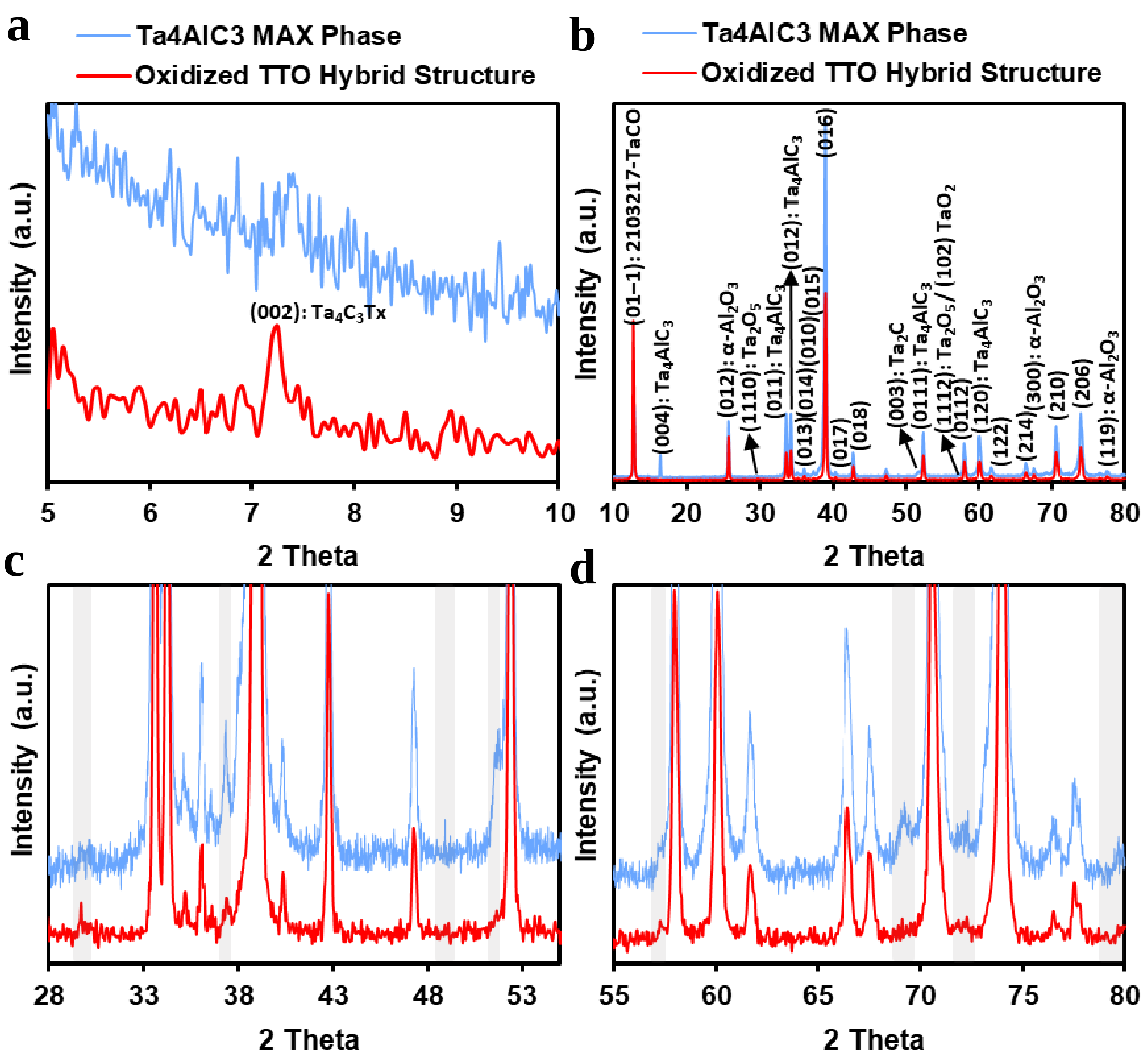

Figure S8: The XRD phase characterization of the Ta_4AlC_3 MAX phase and fluorine-free exfoliated TTO hybrid structure after thermal treatment at 220°C for 2 hours. **(a)** The XRD spectrum of $Ta_4C_3T_x$ MXene nanosheets at 2 Theta = 5 to 10° of (002) present in the structure of TTO hybrid structure. **(b)** The comparison of XRD spectra of the MAX phase and TTO nanostructure at 2 Theta = 10 to 80°. **(c-d)** The XRD analysis of the samples depicted in panels **(c-d)** showed a significant downshift of Al-containing peaks that confirmed the successful synthesis and oxidation of MXene nanosheets. The peaks were matched according to ICSD-156383, 96-210-3218, and α-alumina reference codes.

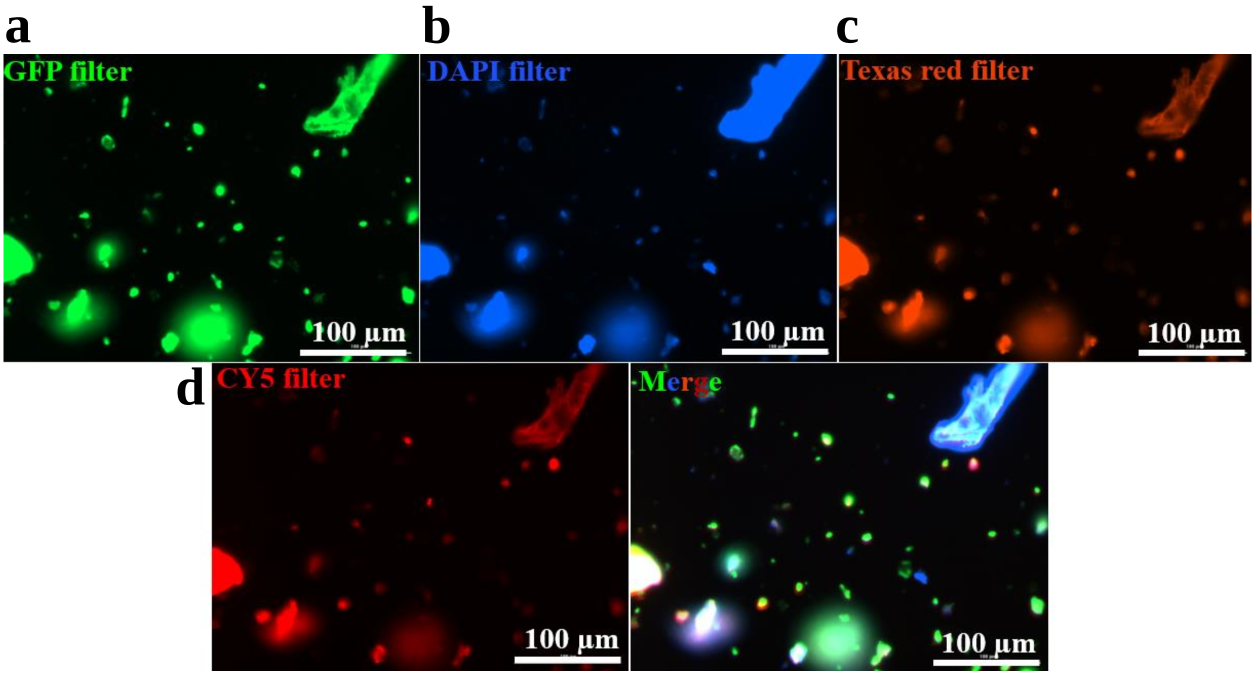

Figure S9: Autofluorescence property of the aqueous TTO hybrid structure at a concentration of 50 µg ml^{-1} was assessed by the BioTek Cytation5 Imaging Multi-Mode Reader at different excitation-emission wavelengths and fluorescence filters: **(a)** GFP (Ex: 469nm/Em: 525nm) **(b)** DAPI (Ex: 377nm/Em: 447nm), **(c)** Texas Red (Ex: 586nm/Em: 647nm), and **(d)** Cy5 (Ex: 628nm/Em: 685nm) filters. The images captured using four different filters were merged (panel bottom right). The aqueous colloidal suspension of TTO at a concertation of 50 µg mL^{-1} exhibited high degrees of autoflorescence at different wavelength regions across the visible spectrum.

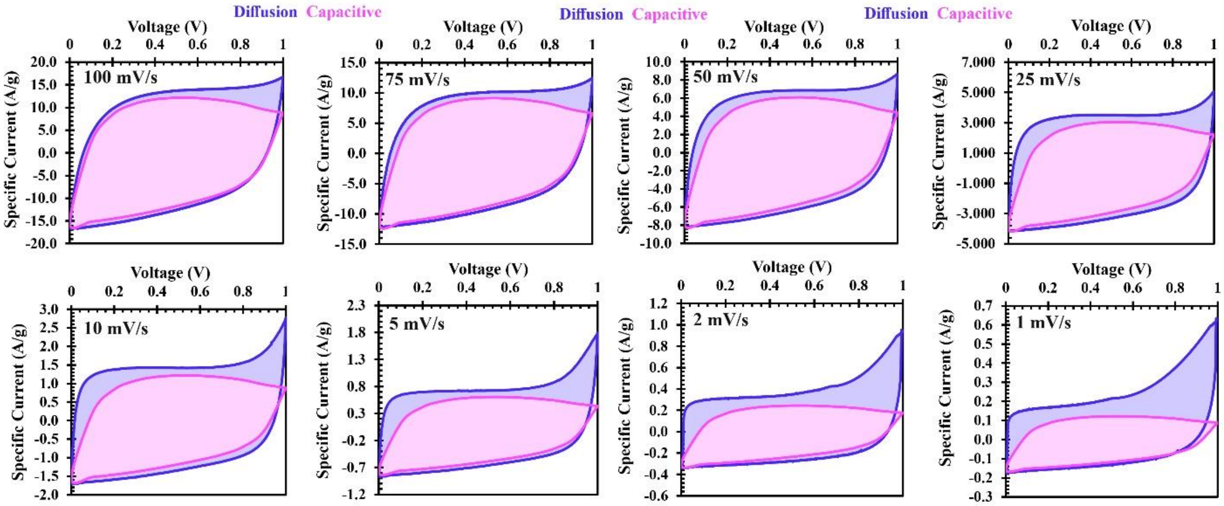

<u>Figure S10:</u> The cyclic voltammetry curves of TTO hybrid structure at different scan rates: 100, 75, 50, 25, 10, 5, 2, and 1 mV/s **(from top left panel to bottom right panel)**. The blue and pink areas show contributions of the diffusion and capacitance mechanisms, respectively. These data show that decoupling result for the TTO hybrid structure electrode at 100 mV s^{-1} offers a remarkable fast-kinetics contribution. Furthermore, the contribution of slow capacitance increased with a decrease in scan rate.

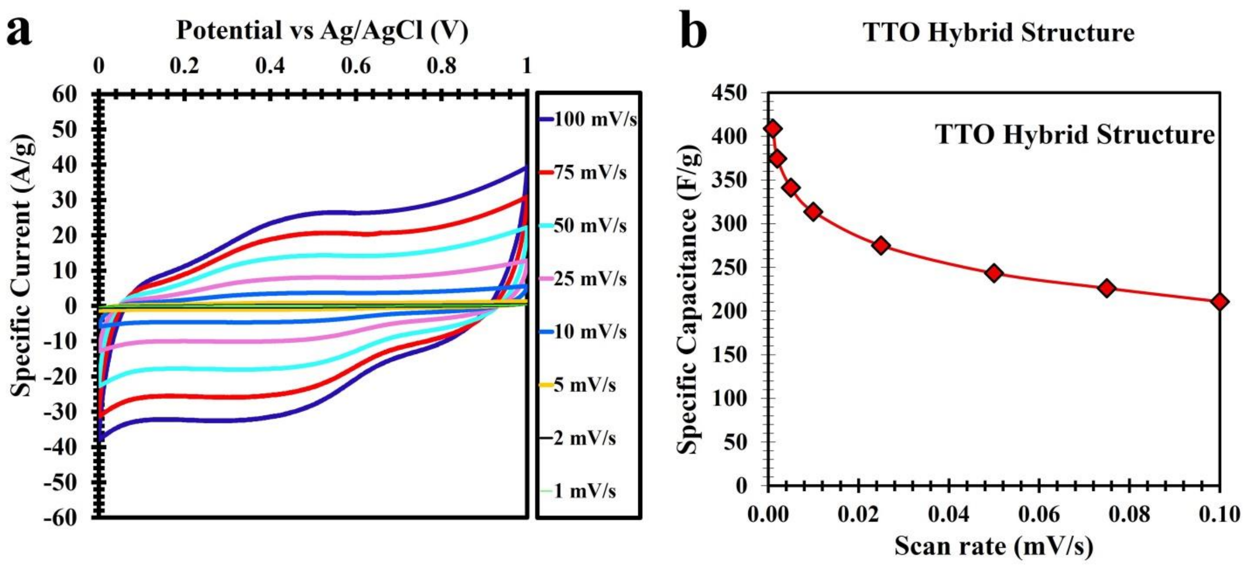

<u>Figure S11:</u> **(a)** Cyclic voltammetry curves, and **(b)** specific capacitance vs. scan rate curve of the TTO electrode were measured using a three-electrode setup in phosphoric acid electrolyte. Our data demonstrate that the obtained results from the three-electrode experiment are in good agreement with the data obtained with solid state two-electrode system. These data further validate the efficacy of the TTO hybrid structure as a biocompatible material for size-sensitive applications.

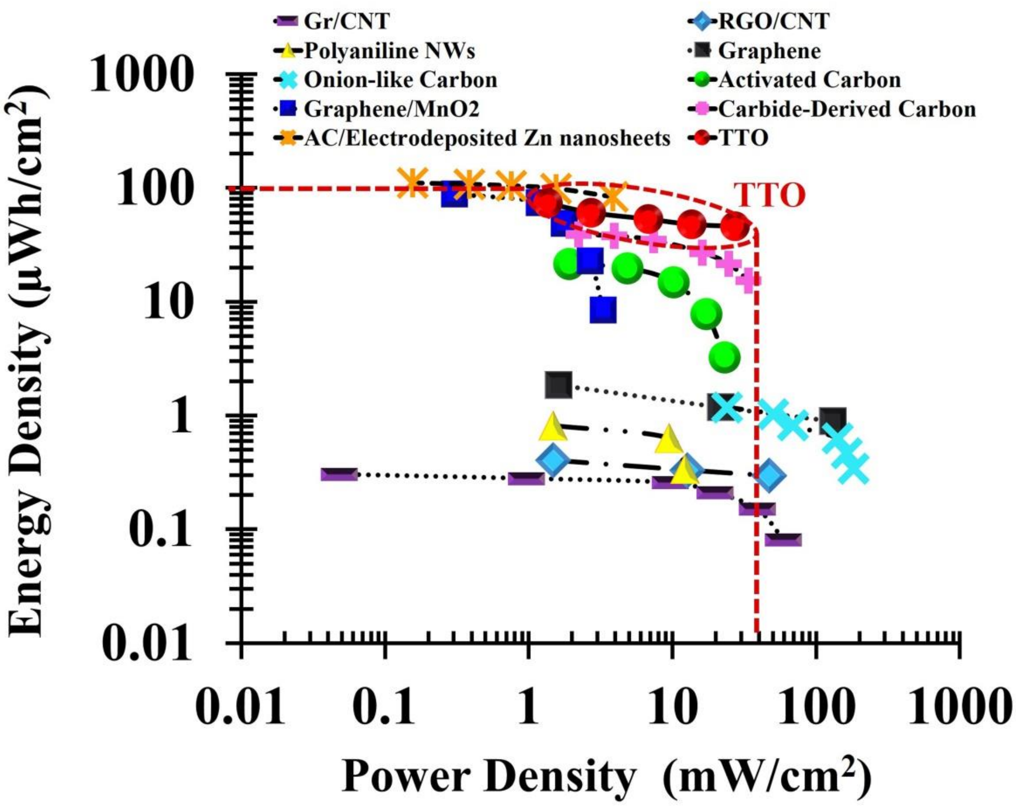

Figure S12: The Ragone plots (areal energy density vs. areal power density) for TTO hybrid structure electrode, graphene/carbon nanofiber (Gr/CNT), reduced graphene oxide/carbon nanotube (RGO-CNT) (RGO/CNT), polyaniline NWs (P-NW), graphene (Gr), onion-like carbon (OLC), activated carbon (AC), Graphene/MnO_2 (Gr/MnO_2), carbide-derived carbon (CDC).[16-22] The mass of electrolyte was not considered for measuring the areal energy and power densities of TTO electrodes. Our data demonstrate that the TTO hybrid structure electrode possesses excellent areal efficiency when compared with most other recently-published supercapacitor electrode materials. This phenomenon might largely be due to the large specific surface area of TTO and the additional Ta-oxides crystals in the composition of functionalized $Ta_4C_3T_x$ MXene during the synthesis process.

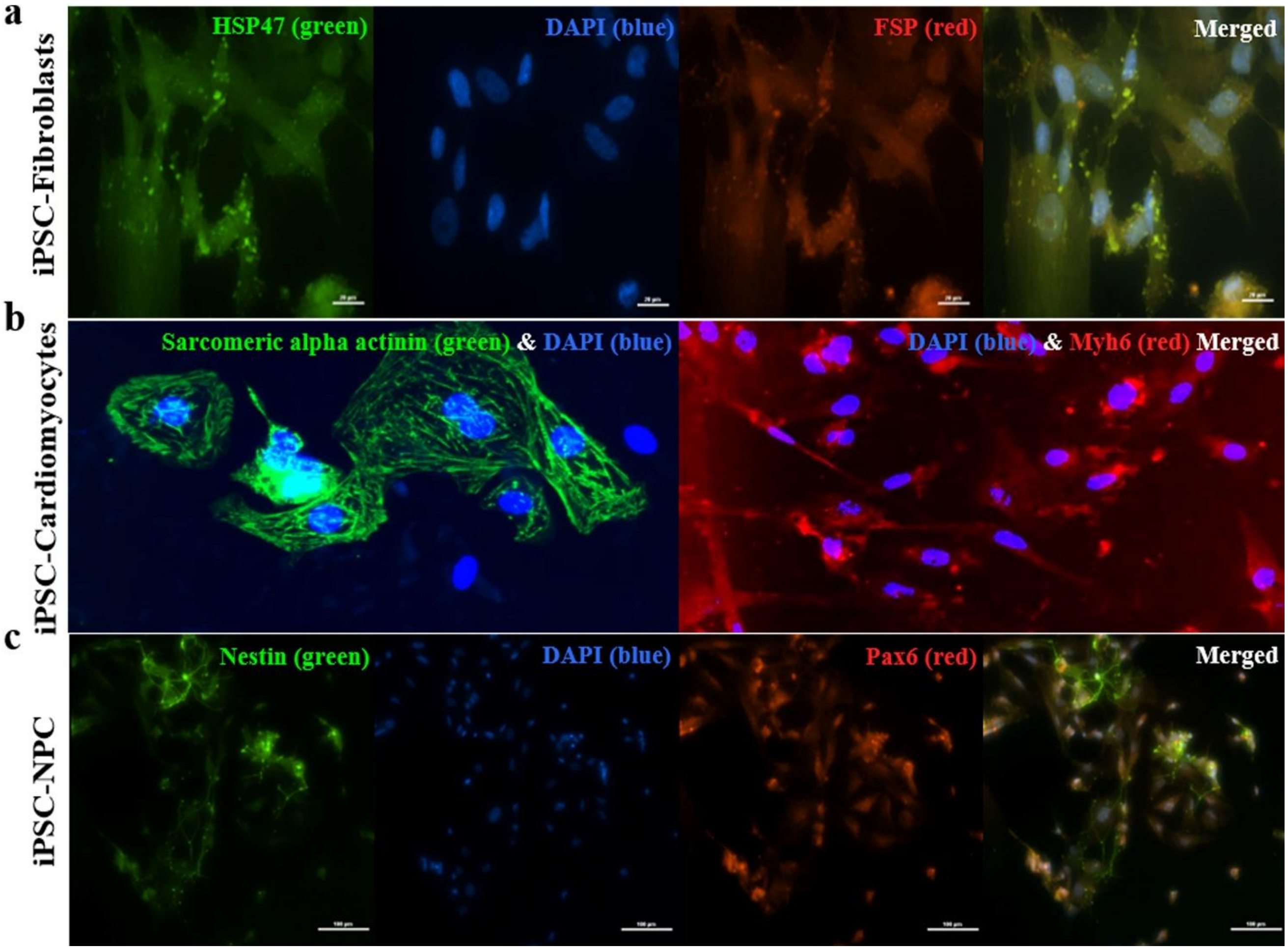

Figure S13: Fluorescence microscopy-based characterization of the human iPSC-derived fibroblasts, cardiomyocytes, and neural progenitor cells. **(a)** Fibroblasts were characterized by immunostaining the cells with HSP47 (green) and FSP (red). **(b)** Cardiomyocytes were characterized by immunostaining the cells with sarcomeric alpha actinin (Green) and Myh6 (red). NPCs were characterized by immunostaining the cells with nestin (Green) and Pax6 (red). DAPI was used to stain nuclei. The images were captured using Cytation5 Imaging system (BioTek Inc.)

References

[1] A. Lamberti, C. F. Pirri, *Journal of Energy Storage* **2016**, *8*, 193.

[2] S. He, Y. Hu, J. Wan, Q. Gao, Y. Wang, S. Xie, L. Qiu, C. Wang, G. Zheng, B. Wang, H. Peng, *Carbon* **2017**, *122*, 162.

[3] H. J. Sim, C. Choi, D. Y. Lee, H. Kim, J.-H. Yun, J. M. Kim, T. M. Kang, R. Ovalle, R. H. Baughman, C. W. Kee, S. J. Kim, *Nano Energy* **2018**, *47*, 385.

[4] J. S. Chae, N.-S. Heo, C. H. Kwak, W.-S. Cho, G. H. Seol, W.-S. Yoon, H.-K. Kim, D. J. Fray, A. T. E. Vilian, Y.-K. Han, Y. S. Huh, K. C. Roh, *Nano Energy* **2017**, *34*, 86.

[5] I. M. Mosa, A. Pattammattel, K. Kadimisetty, P. Pande, M. F. El-Kady, G. W. Bishop, M. Novak, R. B. Kaner, A. K. Basu, C. V. Kumar, J. F. Rusling, *Advanced Energy Materials* **2017**, *7*, 1700358.

[6] C. A. Amarnath, N. Venkatesan, M. Doble, S. N. Sawant, *J. Mater. Chem. B* **2014**, *2*, 5012.

[7] J. Hur, K. Im, S. Hwang, B. Choi, S. Kim, S. Hwang, N. Park, K. Kim, Sci Rep 2013, 3, 1.

[8] M. Salari, S. H. Aboutalebi, I. Ekladious, R. Jalili, K. Konstantinov, H. K. Liu, M. W. Grinstaff, *Advanced Materials Technologies* **2018**, *3*, 1700194.

[9] H. Park, J. W. Kim, S. Y. Hong, G. Lee, D. S. Kim, J. hyun Oh, S. W. Jin, Y. R. Jeong, S. Y. Oh, J. Y. Yun, J. S. Ha, *Advanced Functional Materials 2018*, *28*, 1707013.

[10] J. Yun, C. Song, H. Lee, H. Park, Y. R. Jeong, J. W. Kim, S. W. Jin, S. Y. Oh, L. Sun, G. Zi, J. S. Ha, *Nano Energy* **2018**, *49*, 644.

[11] G. Lee, D. Kim, D. Kim, S. Oh, J. Yun, J. Kim, S.-S. Lee, J. S. Ha, *Energy Environ. Sci.* **2015**, *8*, 1764.

[12] G. Lee, S.-K. Kang, S. M. Won, P. Gutruf, Y. R. Jeong, J. Koo, S.-S. Lee, J. A. Rogers, J. S. Ha, *Advanced Energy Materials* **2017**, *7*, 1700157.

[13] S. R. Mangisetti, M. Kamaraj, R. Sundara, *ACS Omega* **2019**, *4*, 6399.

[14] S. Berchmans, A. J. Bandodkar, W. Jia, J. Ramírez, Y. S. Meng, J. Wang, *J. Mater. Chem. A* **2014**, *2*, 15788.

[15] J. S. Chae, S. K. Park, K. C. Roh, H. S. Park, *Energy Storage Materials* **2019**.

[16] L. Jian, C. Zhang, Z. Yan, Y. Zhu, Z. Peng, R. H. Hauge, D. Natelson, J. M. Tour, *Nano letters* **2013**, 13, 72-78.

[17] W. Lu, N. Nitta, G. Yushin, *Acs Nano* **2013**, 7, 6498-6506.

[18] M. Chuizhou, J. Maeng, S. WM John, P. P. Irazoqui, *Advanced Energy Materials* **2014**, 4, 1301269.

[19] E. MF, R. B. Kaner, *Nature communications* **2013**, 4, 1-9.

[20] P. David, M. Brunet, H. Durou, P. Huang, V. Mochalin, Y. Gogotsi, P. L. Taberna, P. Simon, *Nature nanotechnology* **2010**, 5, 651-654.

[21] E. MF, I. M, Li M, Hwang JY, Mousavi MF, Chaney L, Lech AT, Kaner RB, *Proceedings of the National Academy of Sciences* **2015**, 112, 4233-4238.

[22] P. Huang, C. Lethien, S. Pinaud, K. Brousse, R. Laloo, V. Turq, M. Respaud, A. Demortiere, B. Daffos, P. L. Taberna, B. Chaudret, *Science* **2016**, 351, 691-695.
